# ACP5‐positive macrophages contribute to cerebral oedema and neuroinflammation after traumatic brain injury

**DOI:** 10.1002/ctm2.70845

**Published:** 2026-09-25

**Authors:** Dang Hanhan, He Kun, Zhang Yulian, Zhang Chuanpeng, Zhang Yunsheng, Wang Zixi, Yang Xu, Chen Pengyu, Zhang Li, Yu Yanbing

**Affiliations:** ^1^ China–Japan Friendship Hospital (Institute of Clinical Medical Sciences) Chinese Academy of Medical Sciences & Peking Union Medical College Beijing China; ^2^ Department of Neurosurgery China–Japan Friendship Hospital Beijing China; ^3^ Department of Pain Management China–Japan Friendship Hospital Beijing China; ^4^ Department of Neurology Johns Hopkins School of Medicine Baltimore Maryland USA; ^5^ Peking University China–Japan Friendship School of Clinical Medicine Beijing China

**Keywords:** brain oedema, macrophage, neuroinflammation, secondary injury, traumatic brain injury (TBI)

## Abstract

**Background:**

Traumatic brain injury (TBI) is a leading cause of death and long‐term disability worldwide. Secondary cerebral oedema, driven by neuroinflammation and blood–brain barrier (BBB) disruption, critically determines poor outcomes, yet effective targeted therapies remain absent. This study aimed to identify immune cell subsets associated with post‐TBI cerebral oedema and to explore their functional contributions in preclinical models.

**Methods:**

We integrated single‐cell transcriptomic datasets from a mouse TBI model (GSE175430) and human intracerebral haemorrhage (ICH) perihaematomal oedema samples (GSE266873) with our previously published temporal bulk RNA‐seq profiling data. Through cross‐species comparative analysis, Mfuzz clustering, AUCell scoring and gene set enrichment analysis (GSEA), *Acp5* was identified as an oedema‐associated signature gene, and its macrophage‐enriched expression pattern was supported by single‐cell profiling and immunofluorescence colocalisation. Using the controlled cortical impact (CCI) model, we validated *Acp5* expression dynamics across injury severities. Pharmacological inhibition (AubipyOMe) and genetic knockout of *Acp5* were employed to assess brain oedema, neuroinflammation, neurological function and long‐term cognitive recovery.

**Results:**

A previously unrecognised population of ACP5‐positive monocytes/macrophages infiltrated the brain parenchyma within 1–3 days post‐injury, coinciding with peak oedema severity and neurological deficits. Inhibiting ACP5 significantly reduced brain water content, BBB leakage and proinflammatory cytokines, while improving sensorimotor function. Mechanistically, ACP5‐positive macrophages were associated with CSF2‐related JAK/STAT and NF‐κB signalling. Genetic *Acp5* ablation attenuated acute macrophage infiltration and neuroinflammation, and was associated with improved long‐term behavioural outcomes.

**Conclusions:**

ACP5‐positive macrophages represent an injury‐associated myeloid population that contributes to neuroinflammatory and oedema‐related phenotypes after experimental TBI. Pharmacological and genetic findings provide preclinical evidence supporting further investigation of ACP5 and CSF2‐related signalling in secondary TBI injury.

## Key points

- Integrated transcriptomic analyses identified ACP5‐positive monocytes/macrophages associated with post‐traumatic cerebral oedema and neuroinflammation.
- Pharmacological and genetic targeting of ACP5 attenuated inflammatory phenotypes and improved functional outcomes in experimental TBI.
- CSF2‐related JAK/STAT and NF‐κB signalling may contribute to ACP5‐associated neuroinflammatory responses after TBI.

## Graphical abstract

Integrated transcriptomic analyses identified an ACP5‐positive monocyte/macrophage population enriched in the injured brain within 1–3 days after traumatic brain injury (TBI). Pharmacological and genetic targeting of ACP5 attenuated cerebral oedema and neuroinflammation, preserved blood–brain barrier integrity, and improved long‐term cognitive outcomes. CSF2‐related JAK1/STAT3/NF‐κB signalling was implicated as a candidate downstream pathway.

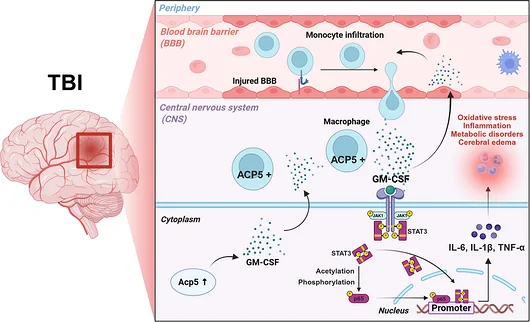

## 1 INTRODUCTION

Traumatic brain injury (TBI) is a serious public health problem caused by external physical forces, resulting in brain damage and neurological dysfunction.^1^ The pathophysiological processes following TBI involve a complex cascade of events, including neuroinflammation, neuronal injury and potential long‐term neurological impairments.^2^, ^3^, ^4^ TBI remains a leading cause of death and long‐term disability worldwide, with secondary brain oedema being a key determinant of adverse clinical outcomes.^5^ Current therapeutic strategies for brain oedema aim to reduce intracranial pressure (ICP) and maintain cerebral perfusion pressure, including the use of osmotic agents such as mannitol and hypertonic saline.^6^ These treatments are nontargeted and can only temporarily lower ICP, but they fail to address the underlying molecular mechanisms associated with blood‒brain barrier (BBB) disruption and have significant side effects.^7^ Notably, the application of traditional osmotherapy in certain patients with severe TBI remains questionable, and there is a considerable risk of ICP rebound effects.^8^, ^9^ Regrettably, despite the emergence of several promising studies focusing on novel targets, there are currently no established targeted therapies addressing the underlying pathophysiology of TBI,^7^, ^10^, ^11^ which highlights the urgent need for new mechanistic insights and targeted interventions.

When the brain suffers mechanical injury, the process is often complex and protracted, involving direct primary injury, subsequent secondary injury and the process of neural repair and regeneration.^12^ In response to acute phases, both resident cells of the central nervous system (CNS) and peripheral inflammatory cells react rapidly, subsequently releasing inflammatory mediators.^13^ This phenomenon has been attributed mainly to CNS‐resident cells such as microglia and astrocytes.^14^ These responses are often referred to as sterile immune responses. The BBB is a critical determinant of TBI outcomes,^15^ as its disruption exacerbates vasogenic oedema, inflammatory infiltration and secondary neuronal damage.^16^ While the breakdown of the BBB following TBI is well documented, the cellular effectors that drive this process remain a subject of contention.^17^, ^18^ Previous studies have indicated that the recruitment of inflammatory cells is an independent pathological process following TBI.^19^ Monocyte‐derived macrophages are key innate immune responders to acute TBI and may play crucial roles in early harmful inflammation and subsequent beneficial repair.^20^, ^21^, ^22^ However, it remains unclear whether specific macrophage subpopulations contribute to acute BBB dysfunction. This knowledge gap hinders the development of targeted therapies for stabilising the neurovascular unit in the early post‐injury phase.

Here, we employed an integrated multiomics approach to elucidate the heterogeneity of peripheral immune infiltration following TBI. Our analysis revealed a distinct ACP5‐positive macrophage subset that preferentially accumulated in the injured brain region within 1–3 days post‐TBI. This subset was closely correlated with the severity of brain oedema and neurological deficits, indicating its potential functional role in the progression of the acute phase of the disease. Notably, targeted intervention for this molecular subgroup alleviated neuroinflammation and improved neurological function, providing preclinical evidence supporting further investigation of ACP5 in acute TBI.

## 2 MATERIALS AND METHODS

### 2.1 Acquisition and processing of scRNA‐seq and bulk RNA‐seq data

We selected the single‐cell datasets GSE175430 and GSE266873, which focus on injured brain tissue from mice following TBI and oedema tissue after haemorrhage in humans, for our study. For dataset GSE175430, researchers employed the controlled cortical impact (CCI) model, categorising subjects into a control group and a TBI group, with *n* = 3 biological samples in each group. The GSE266873 dataset characterised the immune landscape associated with the progression of perihaematomal oedema in humans. This dataset comprises nine samples divided into three groups: Group 1 (*n* = 3), representing time points from 0 to 6 h post‐haemorrhage; Group 2 (*n* = 3), covering the period from 6 to 24 h post‐haemorrhage; and Group 3 (*n* = 3), encompassing time points from 24 to 48 h post‐haemorrhage. The Seurat object was constructed by applying specific filtering criteria: each gene had to be expressed in a minimum of three cells, while each cell needed to express at least 200 genes and no more than 5000 genes. Then, LogNormalise was employed to standardise the expression values. Principal component analysis (PCA) was conducted to achieve linear dimensionality reduction on the standardised expression data. The top 20 principal components were subsequently selected for further analysis. Cell clusters were identified via FindNeighbors and FindClusters with a resolution of  .8. Finally, the uniform manifold approximation and projection (UMAP) algorithm was utilised for visualisation purposes. Leveraging professional expertise, literature and the CellMarker 2.0 database,^23^, ^24^, ^25^, ^26^ we annotated and classified the various cell populations as follows: GSE175430: natural killer (NK) cells (‘*Klrb1c*’, ‘*Ncr1*’), dendritic cells (‘*Flt3*’, ‘*Cd40*’, ‘*Cd80*’), microglia (‘*Tmem119*’, ‘*P2ry12*’, ‘*Cx3cr1*’), endothelial cells (‘*Cldn5*’, ‘*Abcb1a*’, ‘*Flt1*’), macrophages (‘*Ms4a7*’, ‘*Ccr2*’, ‘*Nr4a1*’, ‘*Treml4*’), neutrophils (‘*Lcn2*’, ‘*Ly6g*’), T cells (‘*Cd8a*’, ‘*Cd3e*’) and B cells (‘*Cd19*’, ‘*Cd79a*’); GSE266873: oligodendrocytes (‘*MOG*’, ‘*MBP*’, ‘*PLP1*’), neutrophils (‘*CXCR2*’, ‘*FCGR3B*’), microglia (‘*TREM2*’, ‘*LRMDA*’, ‘*P2RY12*’, ‘*CX3CR1*’), NK/T cells (‘*CD3D*’, ‘*CD3E*’, ‘*NKG7*’), astrocytes (‘*GFAP*’, ‘*AQP4*’). The bulk RNA‐seq time‐course data were derived from our previous study on the atlas of gene expression dynamics in TBI over time—specifically, at sham, TBI‐6 h, TBI‐12 h, TBI‐1 d, TBI‐3 d and TBI‐7 d.^27^

### 2.2 Cerebral oedema score

Candidate genes related to cerebral oedema were initially identified through GeneCards searches using the terms ‘cerebral oedema’, ‘brain oedema’, and ‘traumatic brain injury’. These candidates were further analysed using bulk RNA‐seq time‐course data and Mfuzz clustering. The gene signature was scored using different algorithms according to the data type. AUCell was applied to single‐cell RNA‐seq datasets to estimate signature activity at the individual‐cell level. GSVA was applied to bulk RNA‐seq datasets to generate sample‐level oedema‐associated scores for group comparison and correlation with magnetic resonance imaging (MRI)‐derived oedema volume.

### 2.3 Identification of differentially expressed genes and enrichment analysis

For scRNA‐seq, differential expression analysis was conducted via the ‘FindMarkers’ package, whereas bulk RNA‐seq data were analysed via the ‘DESeq2’ package. In both analyses, genes meeting the criteria of an adjusted *p* value <.05 and an absolute log2(fold change) >1.5 were identified as differentially expressed genes (DEGs). Gene Ontology (GO) analysis and Kyoto Encyclopedia of Genes and Genomes (KEGG) pathway analysis were subsequently conducted via a hypergeometric distribution algorithm to screen for significantly enriched functions and signalling pathways via the R package ‘clusterProfiler’.^28^ In addition, we used ‘c2.cp.kegg.v7.4.symbols.gmt’ and ‘c5.go.v7.4.symbols.gmt’ for further gene set enrichment analysis (GSEA).^29^

### 2.4 Pseudotime trajectory inference

Pseudotime trajectory analysis was performed using Monocle 2 with the DDRTree dimensionality reduction method. Cell trajectories and density distributions were visualised using ggplot2.

### 2.5 Cell interaction analysis

CellChat was utilised to infer and visualise the interactions among cells. First, the differentially expressed ligands and receptors for each cell group were identified via the ‘cellchatdb. human’ or ‘cellchatdb. mouse’ database. By calculating the intensity of cell communication—such as the probability of ligand‒receptor binding—the communication strength between various cell types can be quantitatively analysed.

### 2.6 Animals and cells

To eliminate the potential confounding effects of oestrogen on TBI outcomes, this study utilised male mice exclusively.^30^ Adult male C57BL/6 mice (10 weeks old, 23–25 g) and *Acp5* knockout (*Acp5‐*KO) mice (C57BL/*^6JGpt–Acp5em24Cd10178^*/Gpt, 10 weeks old, 23–25 g) were purchased from GemPharmatech Co., Ltd. Mice were maintained under specific‐pathogen‐free conditions on a 12‐h light/dark cycle with ad libitum access to food and water. The experimental procedures were performed in accordance with the Guide for the Care and Use of Laboratory Animals published by the National Institutes of Health and the ARRIVE Guidelines 2.0 (*Animal Research: Reporting of In Vivo Experiments*)^31^ and were approved by the Institutional Animal Experimental Committee. The immortalised RAW264.7 cells were purchased from Pricella Biotechnology Co., Ltd. and cultured in Dulbecco's modified Eagle's medium (DMEM; Gibco) supplemented with 10% foetal bovine serum (Gibco) and 1% penicillin‒streptomycin solution (Wuhan Pricella Biotechnology Co., Ltd.) according to the product instructions. The cells were cultured in a 5% CO_2_ incubator at 37°C.

### 2.7 TBI model

In accordance with previous studies,^32^, ^33^ TBI was induced via the CCI model. Briefly, after anaesthesia with intraperitoneal (i.p.) injection of 1% sodium pentobarbital (50 mg/kg), the mice were placed in a prone position on a stereotaxic apparatus, ensuring that the head remained level, followed by hair removal and disinfection of the surgical area with iodophor. The skull was fully exposed, and a portable cranial drill was used to create a 4‐mm‐diameter window in the left parietal lobe, positioned 2 mm lateral to the midline, between the bregma and lambda. The parameters of the CCI device (RWD) were configured to achieve an impact speed of 3.5 m/s, a dwell time of 500 ms and tissue depths of 1 mm for mild TBI and 2 mm for severe TBI. Cortical impact was delivered using a 3‐mm‐diameter impactor tip positioned perpendicular to the brain surface. The scalp was sutured immediately after TBI. Animals in the sham group underwent the same surgical procedures (including anaesthesia, scalp incision and craniotomy) but were not subjected to cortical impact, followed by identical scalp closure. Throughout the surgery, a thermostatic heating pad was used to maintain the core body temperature of the animals at 37°C. Mice were randomly assigned to experimental groups. All analyses were performed with the experimenter blinded to group identity.

### 2.8 Pharmacological inhibition of ACP5

AubipyOMe (a small‐molecule inhibitor of ACP5, Sigma, SML2210) was dissolved in dimethyl sulfoxide (DMSO) to prepare a 25 mg/mL stock solution, which was then diluted with sterile physiological saline. Mice were randomly assigned to three groups: sham, TBI + vehicle and TBI + AubipyOMe. (1) Sham group did not receive injections. (2) TBI + vehicle group received an equal volume of 2.5% DMSO in saline via i.p. injection immediately, 24 h, and 48 h post‐TBI (a total of three doses). (3) TBI + AubipyOMe group received i.p. injections of AubipyOMe at the same time points.^34^, ^35^ For the TBI groups, each mouse received an i.p. injection of 200 µL of the diluted solution, corresponding to an approximate dose of 5 mg/kg body weight. To characterise the pharmacological activity and cytotoxicity of AubipyOMe in vitro, RAW264.7 cells were treated with 0, 1, 2, 5, 10, 20 or 50 µM AubipyOMe for 24 h, and cell viability was assessed using a CCK‐8 assay. *Acp5* mRNA and ACP5 protein abundance were evaluated by reverse transcription quantitative polymerase chain reaction (RT‐qPCR) and western blotting, respectively, after treatment with 0, 1, 2, 5, 10 or 20 µM AubipyOMe. ACP5 enzymatic activity was measured using a tartrate‐resistant acid phosphatase assay kit (P0332, Beyotime) according to the manufacturer's instructions.

To assess delayed systemic tolerability following short‐term AubipyOMe administration, an independent safety experiment was conducted in uninjured wild‐type (WT) mice. Mice were randomly assigned to received i.p. injections of AubipyOMe according to the same schedule described above. Blood and major organs were collected 28 days after the first administration. Complete blood counts were performed to evaluate leukocyte‐, erythrocyte‐ and platelet‐related parameters. Serum alanine aminotransferase (ALT), aspartate aminotransferase (AST), total protein, blood urea nitrogen (BUN) and creatinine (CREA) levels were measured to assess hepatic and renal function. The brain, heart, liver, spleen, lung and kidney were collected, fixed in 4% paraformaldehyde (PFA), embedded in paraffin, sectioned and stained with haematoxylin and eosin (H&E). Representative tissue sections were examined for overt histopathological alterations.

### 2.9 In vivo recombinant mouse CSF2 rescue experiment

Mice were randomly assigned to four groups: WT‐sham, WT‐TBI, *Acp5*‐KO‐TBI and *Acp5*‐KO‐TBI + CSF2 (*n* = 5 per group). Recombinant mouse CSF2 (P7361, MedChemExpress) prepared in phosphate‐buffered saline (PBS) was administered intraperitoneally at 10 µg/kg immediately after TBI and again at 24 and 48 h post‐injury. The corresponding control groups received an equal volume of vehicle according to the same schedule. Perilesional brain tissue was collected at 3 days post‐injury for western blot analysis.

### 2.10 Mouse brain tissue acquisition

The mice were deeply anaesthetised with 1% pentobarbital sodium (100 mg/kg) and subsequently secured in a stereotactic frame. Next, they were then transcardially perfused with precooled  .9% physiological saline until the liver appeared white. The brain tissue surrounding the injury site (approximately 5 mm × 5 mm × 3 mm) was then harvested for subsequent experiments.

### 2.11 RNA isolation and RNA sequencing

Total RNA from injured and surrounding tissues was isolated using TRIzol reagent (Invitrogen) following the manufacturer's instructions. The purity and concentration of the RNA were assessed via a NanoDrop 2000 spectrophotometer (Thermo Fisher Scientific). Transcriptome sequencing was performed by OE Biotech Co., Ltd. In addition, total RNA from cells was extracted via an RNeasy Animal RNA Isolation Kit with a Spin Column (Beyotime).

### 2.12 Neuroinflammation model induced by lipopolysaccharide

RAW264.7 cells were cultured in 12‐well plates with approximately 2 × 10^5^ cells per well. When the cell confluence reached approximately 80%, 100 ng/mL lipopolysaccharide (LPS) diluted in medium was added for further culture, and total RNA was then extracted after 12 h of stimulation for subsequent experiments.

### 2.13 Transfection of small interfering RNA

Small interfering RNA (siRNA) silencing was used to explore the molecular mechanisms of genes. The target *Acp5* sequence of the siRNA was GCTACTTGCGGTTTCACTA, which was provided by TsingKe Biological Technology. Negative control siRNA (siNC) and *Acp5* siRNA (si*Acp5*) were transfected into cells via Opti‐MEM containing Lipofectamine RNAiMAX (Invitrogen). The specific procedure was as follows: Opti‐MEM containing RNAiMAX and siRNA was mixed thoroughly, allowed to stand at room temperature for 20 min, and then added to the culture medium. After 4 h of transfection, the transfection medium was replaced with fresh DMEM and cultured for an additional 24 h to evaluate the knockdown effect of *Acp5*. The cells were subsequently used for the indicated experiments.

### 2.14 Transwell assay

Cell migration was analysed via a Transwell assay as described previously.^36^ Briefly, the upper and lower chambers were separated by a polycarbonate filter membrane with a pore size of 8 µm (Corning, Inc.). The upper chamber was filled with serum‐free medium (DMEM), and the lower chamber was filled with medium containing 10% FBS and LPS (100 ng/mL). The transfected RAW264.7 cells were subsequently divided into two groups, the LPS + siNC group and the LPS + si*Acp5* group, and seeded in the lower chamber (10^5^ cells/mL). Untransfected RAW264.7 cells (5 × 10^4^ cells/mL) were seeded in the upper chamber. Both chambers were incubated at 37°C for 24 h. After that, the surface of the membrane was fixed with 4% PFA at room temperature for 30 min and wiped with a cotton swab, and the cells that migrated to the lower surface of the membrane were stained with crystal violet solution. Finally, migrated cells were counted in five randomly selected fields per well under a 20× bright‐field microscope.

### 2.15 Brain water content

To evaluate the extent of brain oedema following TBI, we measured the brain water content via the dry‐wet weight method.^37^, ^38^ The ipsilateral and contralateral cerebral hemispheres were carefully separated, excluding the olfactory bulb and cerebellum. Each hemisphere was immediately weighed, and the wet weight was recorded. The tissues were subsequently dried at 110°C for 48 h until a constant weight was achieved, after which they were weighed again to obtain the dry weight. The brain water content was calculated via the following formula: (1 − dry weight/wet weight) × 100%. The final assessment of brain oedema was determined by calculating the percentage increase in water content on the ipsilateral (injured) side as follows: [(ipsilateral brain water content percentage/contral brain water content percentage) − 1] × 100%.

### 2.16 BBB permeability assays

BBB permeability was assessed via Evans blue staining.^37^ A 2.0% solution of Evans blue (2 mL/kg) was administered via tail vein injection. Following a circulation period of 1 h, the eyes, limbs and tails of the mice exhibited blue colouration. The mice were subsequently perfused with PBS to remove any residual dye. The ipsilateral cerebral hemispheres were meticulously dissected and homogenised in PBS. The resulting homogenate was centrifuged to obtain the supernatant, to which an equal volume of trichloroacetic acid was added. The samples were then incubated at 4°C for 18–24 h to precipitate proteins and extract the dye. After incubation, the absorbance of the supernatant was measured at 620 nm via a spectrophotometer to quantify the extravasated dye.

### 2.17 MRI analysis

To further assess the extent of brain injury and oedema, a small animal 3 T MRI scanner was used for T2‐weighted imaging.^39^ For volumetric quantitative analysis, T2‐weighted images were analysed. A researcher blinded to the experimental groups manually delineated the hyperintense regions (indicative of vasogenic oedema and tissue damage) on each consecutive slice using ImageJ software (version 1.54f).^40^ The total oedema volume was calculated by summing the delineated areas and multiplying by the slice thickness. To account for potential individual differences in brain size, the oedema volume was normalised and expressed as a percentage of the ipsilateral hemispheric volume (oedema volume ratio = [oedema volume / hemispheric volume] × 100%). All image acquisitions and analyses were conducted using identical parameters to ensure consistency.

### 2.18 Reverse transcription quantitative polymerase chain reaction

NovoScript^®^Plus All‐in‐one 1st Strand cDNA Synthesis SuperMix (Novoprotein Scientific Inc.) was used to efficiently synthesise first‐strand cDNA using total RNA as a template. Then, NovoStart^®^ Fast SYBR qPCR SuperMix (Novoprotein Scientific Inc.) was prepared for RT‐qPCR via a QuantStudio 5 Real‐Time PCR System (Thermo Fisher Scientific). The experiment was conducted in strict accordance with the manufacturer's instructions. The primers used in the study were designed independently and synthesised by TsingKe Biological Technology and are displayed in Table S1. All the experiments included three biological replicates. The relative mRNA expression levels were normalised to those of *Gapdh*, which was used as a reference gene, via the 2^−ΔΔCq^ method.^41^

### 2.19 Western blot analysis

The samples were homogenised in ice‐cold lysis buffer containing protease and phosphatase inhibitors (MCE). The supernatants were collected, and the protein concentration was determined via a BCA protein assay kit (Proteintech). Equal amounts of protein were separated by 10% SDS‒PAGE and subsequently transferred to a  .45 µm polyvinylidene fluoride (PVDF) membrane (Millipore). The membrane was blocked with rapid blocking solution (Beyotime) at room temperature for 30 min and then incubated overnight at 4°C with specific primary antibodies. The primary antibodies used included anti‐ACP5 (1:1000, F2037, Selleck), anti‐OCCLUDIN (1:1000, A5381, Selleck), anti‐CSF2 (1:1000, PA2949, Abmart), anti‐JAK1 (1:1000, TA5012, Abmart), anti‐p‐JAK1 (1:1000, TA2012, Abmart), anti‐p‐STAT3 (1:1000, T56566, Abmart), anti‐NF‐κB‐p65 (1:1000, F0006, Selleck), anti‐p‐NF‐κB‐p65 (1:1000, 3033, CST), anti‐α‐TUBULIN (1:1000, HRP‐66031, Proteintech) and anti‐VINCULIN (1:1000, F0110, Selleck) antibodies. Following this incubation period, the membrane was incubated with the corresponding horseradish peroxidase (HRP)‐conjugated secondary antibodies at room temperature for 1 h. The secondary antibodies used included HRP‐conjugated goat anti‐rabbit IgG (1:10 000, SA00001‐2, Proteintech) and HRP‐conjugated goat anti‐mouse IgG (1:10 000, SA00001‐1, Proteintech). The protein bands were visualised via a gel imaging system (Bio‐Rad) and analysed with ImageJ software (version 1.54f).

### 2.20 Enzyme‐linked immunosorbent assay

On the third day after TBI, the level of CSF2 in the brain tissue was determined via an enzyme‐linked immunosorbent assay (ELISA) kit from Beijing Bio Wok Technology according to the manufacturer's instructions.

### 2.21 H&E staining

The tissue samples were fixed in 4% PFA (Bioshark) at a volume ratio of 1:10, embedded in paraffin and sectioned into 4‐µm slices. The slices were dewaxed with xylene, dehydrated through an ethanol gradient and rinsed with distilled water. The sections were subsequently stained with Harris haematoxylin for 4 min and washed with water until no excess staining solution remained on the sections. Next, differentiation was performed using  .8% acidic alcohol. The sections were subsequently stained with eosin for 20 s without further washing with water, dehydrated with absolute ethanol, cleared with xylene, and mounted for microscopic examination.

### 2.22 Immunofluorescence staining

After the tissue sections were deparaffinised and rehydrated, antigen retrieval was conducted via an ethylenediaminetetraacetic acid (EDTA) antigen retrieval solution. The sections were subsequently blocked with 5% bovine serum albumin (BSA) at room temperature for 1 h and then incubated overnight at 4°C with the primary antibody. The primary antibodies utilised were anti‐ACP5 (1:500, 1070, Novus), anti‐F4/80 (1:500, 70076, CST) and anti‐IBA1 (1:500, OB‐PGP049‐01, BioFarm). The following day, after the sections were rewarmed, they were washed three times with TBST and subsequently incubated with fluorescence‐conjugated secondary antibodies at room temperature for 1 h. The secondary antibodies used included donkey anti‐mouse IgG (1:200, A21203, Thermo Fisher), donkey anti‐rabbit IgG (1:200, A21206, A21207, Thermo Fisher) and goat anti‐guinea pig IgG (1:500, G‐GP488, BioFarm). After washing, nuclear staining was performed via incubation with 4′,6‐diamidino‐2‐phenylindole (DAPI) working solution in the dark at room temperature for 5 min. The sections were then mounted with fluorescence mounting medium and stored at 4°C in darkness until examination under a microscope. Data analysis was carried out via ImageJ software (version 1.54f).

### 2.23 Flow cytometry

The mice were euthanised, and physiological saline was perfused to clear the circulating blood. The brain was immediately excised and immersed in cold Hank's balanced salt solution (HBSS). Subsequently, a block of brain tissue encompassing the injury site (approximately 5 mm × 5 mm × 3 mm) was harvested for further analysis. The collected brain tissue was subjected to enzymatic digestion via a specialised kit for brain tissue dissociation (RWD). The resulting digested suspension of brain tissue was filtered through 70‐µm nylon mesh to obtain a single‐cell suspension. Red blood cells were lysed with red blood cell lysis buffer (Solarbio). On average, 1 × 10^6^ cells were collected from each sample. Following this procedure, the cells were incubated sequentially with specific antibodies^20^: (1) Zombie UV™ Fixable Viability Kit (1:200, Biolegend, 423107) for distinguishing live cells; (2) anti‐mouse CD16/CD32 (1:200, Biolegend, 156604) for blocking Fc receptors; and (3) PE rat anti‐mouse Ly6G (1:200, BD, 551461), Brilliant Violet 421™ anti‐mouse CD11b (1:200, Biolegend, 101235), FITC anti‐mouse CD45 (1:200, Biolegend, 103108) and APC anti‐mouse F4/80 Antibodies (1:200, Biolegend, 100 µg; 123116). The samples were incubated with antibodies on ice for 30 min before being washed three times with PBS. The samples were then resuspended in 100 µL of flow cytometry buffer for subsequent analysis via flow cytometry. Data acquisition was performed via a Beckman flow cytometer, followed by analysis via FlowJo software.

### 2.24 Neurological severity score

The neurological severity score (NSS) assesses overall neurological function through four aspects: behaviour, alertness, balance and motor functions.^42^, ^43^ On the third day after TBI, the NSS of each group of mice was evaluated. A score of 0 indicates the least severe injury, whereas a score of 10 indicates the greatest deficit. To reduce the rater bias, the specific group allocation of the experimental animals was blinded.

### 2.25 Rotarod test

The rotarod test is employed to evaluate the motor coordination abilities of mice.^44^ Prior to the formal assessment, the mice were subjected to a training regimen for five consecutive days, with three sessions each day, to ensure their familiarity with the testing apparatus and their ability to remain on the rod for 3–5 min at an elevated speed. During the formal test, each mouse was required to stay on the rotarod for a duration of 300 s, during which the rotation speed increased linearly from 4 revolutions per minute (rpm) to 40 rpm. The time taken for each mouse to fall off the rod was recorded as the latency to fall. Each mouse was tested every 15 min, and the average latency of the three trials was taken as the final latency to fall.

### 2.26 Wire grip test

The apparatus used was a 50‐cm‐long, 2‐mm‐diameter metal wire suspended between two vertical wooden sticks positioned 40 cm above the ground.^42^, ^45^ The mice were trained to grasp the wire with their front paws and maintain their body weight for at least 60 s before the experiment. A soft surface was placed beneath the wire to prevent injury. During testing, the mice were positioned in the middle of the wire, and the latency to fall was recorded, with a maximum time of 60 s. Each mouse underwent three trials with a 5–10 min inter‐trial interval, and the average latency time was calculated. The final latency was multiplied by each mouse's weight (g) to account for body weight differences.

### 2.27 Beam walking test

The device comprises a wooden balance beam with a diameter of 12 mm and a length of 1 meter, accompanied by a black box and a stand that is 50 cm in height. A light source serves as the stimulus to encourage mice to traverse the balance beam and enter the black box at the opposite end, driven by their instinctual behaviours of approach and avoidance. The duration required for the mice to cross the entire length of the balance beam was recorded.^46^ The mice were trained for 3 days before the surgery, and the assessment was conducted at 3‐day post‐TBI. All the experimenters were unaware of the experimental group.

### 2.28 Morris water maze

In the Morris water maze (MWM) test,^47^ the mice were placed in a 120‐cm‐diameter circular water maze. The water temperature was maintained at 19–22°C, and the water was made opaque with white titanium dioxide and changed daily. The maze was divided into four quadrants, with a 10‐cm‐diameter platform located in the centre of one quadrant, which was submerged 1 cm below the surface. Visual cues of different shapes and colours were placed around the pool walls as spatial references. During the 5‐day training period, each mouse underwent three daily trials, which were given 60 s to locate the platform. If unsuccessful, the mouse was guided to the platform and left there for 30 s. Twenty‐four hours after training, a 60‐s probe test was conducted with the platform removed to assess spatial memory. Swimming speed was recorded and analysed to exclude the possibility that differences in spatial learning and memory were confounded by alterations in motor function. Swimming trajectories were recorded by an overhead camera and analysed automatically via the LabMaze animal behaviour analysis system (Zhongshidichuang Science and Technology).

### 2.29 Open field test

The mice were subjected to an open field test.^42^ The open field apparatus consisted of a black, opaque, acrylic, uncovered square enclosure measuring 50 cm × 50 cm × 35 cm (length × width × height) and was designed to be nontransparent and nonreflective. A digital camera was mounted directly above the apparatus to record the locomotor trajectories of the mice, with its field of view encompassing the entire open field area. The open field arena was subdivided into a central zone and a peripheral zone. The testing environment was maintained under dim illumination and kept free from external noise. Prior to each trial, any residual urine or faeces in the arena were thoroughly cleaned via paper towels moistened with alcohol. At the beginning of each test session, each mouse was gently placed in the lower‐left corner of the open field and allowed to explore freely for a 5‐min period, during which its behaviour and movement trajectories were recorded. The behavioural data were subsequently analysed via the LabMaze animal behaviour analysis system to quantify various parameters, including the time spent in and distance travelled within the central and peripheral zones, followed by comprehensive statistical analysis.

### 2.30 Statistical analysis

All statistical analyses were performed using GraphPad Prism version 9.5.1. For in vivo experiments, *n* represents the number of independent animals, whereas for in vitro experiments, *n* represents independent biological experiments. Data are presented as the mean ± standard deviation. Normality was assessed using the Shapiro–Wilk test. Comparisons between two groups were performed using a two‐sided unpaired Student's *t*‐test for normally distributed data or the Mann–Whitney *U*‐test for non‐normally distributed data. Comparisons among three or more groups were performed using one‐way ANOVA followed by Dunnett's multiple‐comparisons test. MWM acquisition data were analysed using two‐way repeated‐measures ANOVA, followed by Dunnett's multiple‐comparisons test comparing each group with the WT‐TBI group at each training day. Statistical significance was defined as *p* < .05. Exact *p* values are reported whenever applicable; values below  .0001 are reported as *p* < .0001.

## 3 RESULTS

### 3.1 Identification of cerebral oedema‐associated genes and temporal expression dynamics via bulk RNA‐seq

Ultimately, we identified a gene set related to brain oedema (Figure S1), whose functions were further characterised through GO enrichment analysis (Figure S2). We subsequently leveraged bulk RNA‐seq and Mfuzz clustering analysis of gene expression dynamics in TBI mice over time—specifically in the sham, TBI‐6 h, TBI‐12 h, TBI‐1 d, TBI‐3 d and TBI‐7 d groups—from our previous study^27^; we delineated two distinct clusters of genes exhibiting different expression patterns. Notably, Cluster 2 demonstrated significant temporal dynamic changes, with peak expression observed at 3 days post‐TBI. The GO enrichment functions associated with this cluster were linked to processes such as ‘regulation of vascular permeability’, ‘tissue homeostasis’, ‘response to mechanical stimulus’, and ‘maintenance of the blood‒brain barrier’ (Figure S3). Thus, we identified 97 genes from Cluster 2 whose expression dynamics and functional annotations were strongly associated with brain oedema. Leveraging this bioinformatic evidence, we hypothesised that these genes contribute to the pathophysiology of oedema. Notably, the final 97‐gene signature included genes directly related to water transport and ion homeostasis, such as *Aqp4*, *Aqp9*, *Slc12a2*, *Atp1a2*, *Trpm4* and *Trpv4*, as well as genes associated with BBB integrity and vascular function, including *Cldn5*, *Ocln*, *Tjp1*, *Tjp2*, *Esam*, and *Angpt1*.

To determine whether the bulk RNA‐seq‐derived oedema‐associated score reflected anatomically measurable oedema, T2‐weighted MRI was performed before tissue collection for RNA sequencing in the same cohort. MRI‐derived oedema volumes and GSVA scores were matched on an individual‐animal basis (Sham, *n* = 6; TBI, *n* = 10). The GSVA score was significantly increased in TBI mice compared with sham mice (Figure S4A, *p* < .0001). Among TBI mice, animals with scores above the median exhibited significantly greater MRI‐derived oedema volumes than animals with scores below the median (Figure S4B,C, *p* = .0262). Representative T2‐weighted MRI images of sham, low‐score TBI and high‐score TBI animals are shown in Figure S5, and the corresponding 97‐gene expression heatmap for the same animals is provided in Figure S6. Importantly, GSVA scores were strongly positively correlated with MRI‐derived oedema volumes across matched animals (*r* = .8554, *p* = .0016; Figure S7). These findings support the score as an imaging‐correlated molecular signature of post‐TBI oedema‐associated biology.

### 3.2 Single‐cell RNA sequencing revealed significantly increased cerebral oedema scores at 3 days post‐TBI

**FIGURE 1:**
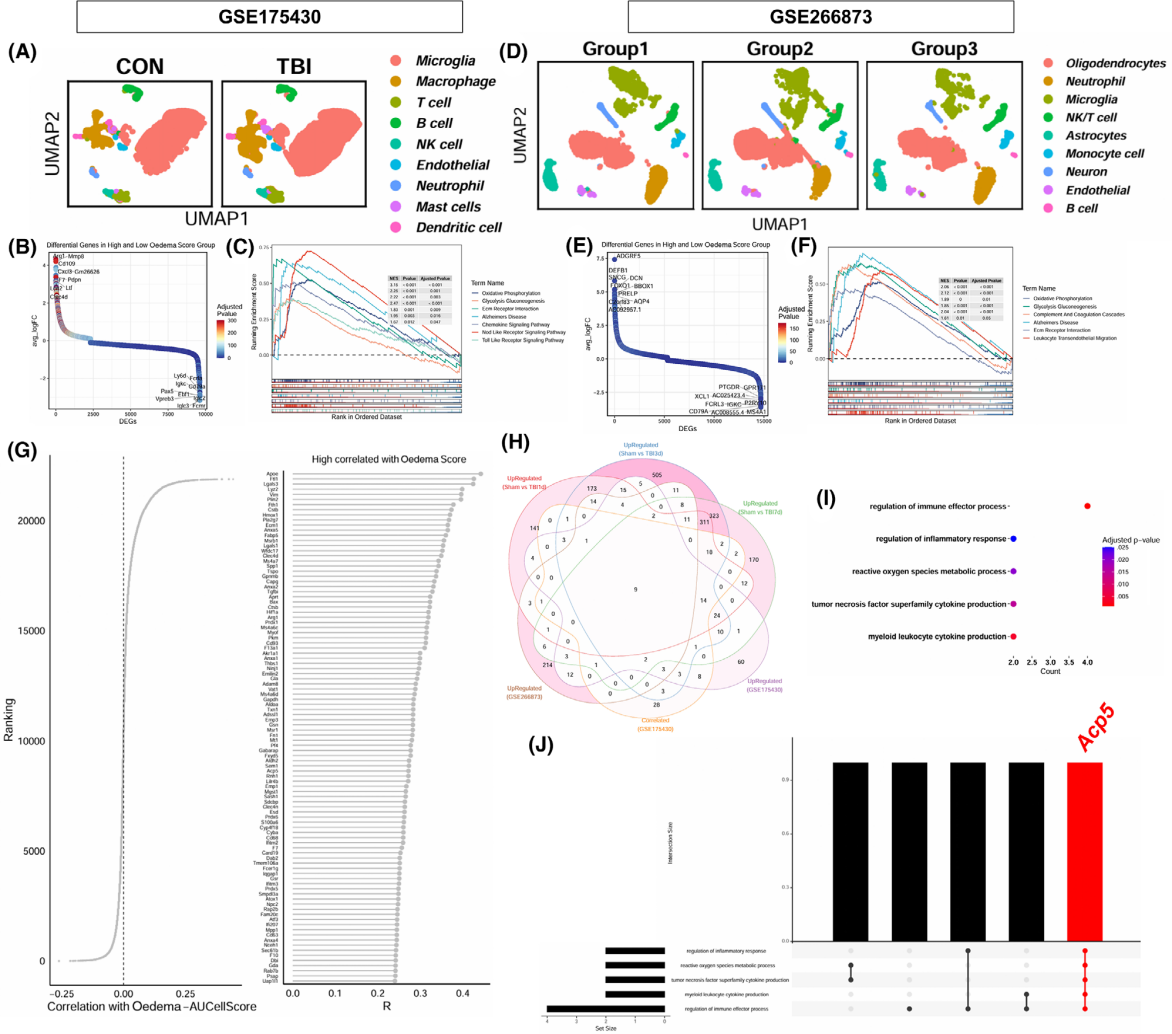
Single‐cell RNA sequencing revealed significantly increased cerebral oedema scores at 3 days post‐traumatic brain injury (TBI). (A) Uniform manifold approximation and projection (UMAP) plot showing the classification of 71 660 cells from CON and TBI mouse brains into major cell types. (B) Volcano plot of differentially expressed genes (DEGs) between high‐ and low‐oedema‐score cells in the TBI group. The top 10 upregulated and downregulated genes are also presented. (C) Gene set enrichment analysis (GSEA) showing significant activation of metabolic and inflammatory pathways in cells with high oedema scores. (D) UMAP plot of cell types in human intracerebral haemorrhage (ICH) perihaematomal oedema samples. (E) Volcano plot of DEGs between high‐ and low‐oedema‐score cells within the human data. The top 10 upregulated and downregulated genes are also presented. (F) GSEA showing significantly activated pathways in human high‐oedema score cells. (G) Pearson correlation analysis of the GSE175430 dataset identified the top 100 genes correlated with brain oedema scores. (H) Venn diagram intersection of upregulated genes from GSE175430 and GSE266873 (high oedema scores with low oedema scores), highly correlated genes with oedema scores from GSE175430, and upregulated genes from bulk RNA‐seq at 1, 3 and 7 days post‐TBI. (I) Gene Ontology (GO) enrichment analysis of the nine intersection genes revealed a significant association with the regulation of inflammation and immunity. (J) UpSet plot with all significantly enriched pathways from (I).

To investigate brain oedema following TBI, we analysed the mouse single‐cell dataset GSE175430 alongside our previously published bulk RNA‐seq data. The single‐cell dataset comprised 71 660 isolated cells from six samples across two groups, which were classified into several cell types: dendritic cells, endothelial cells, microglia, macrophages, neutrophils, NK cells, T cells and B cells (Figures S9 and 1A). AUCell analysis showed that the oedema‐associated score was significantly higher in the TBI group than in the control group (Figure S11). Evaluation of the oedema score across cell types after TBI revealed a marked increase in all categories (Figure S11). Specifically, 62.53% of the cells in the TBI group presented a high oedema‐score category, compared with only 34.15% in the control group (Figure S12). These observations were corroborated by Mfuzz time series clustering analysis, which demonstrated sustained temporal activation in gene modules associated with oedema progression. GSVA of bulk RNA‐seq data further indicated that the activity of the oedema‐associated transcriptional signature peaked at this time point (Figure S8). To explore functional differences between cells with high and low oedema scores, we re‐evaluated the TBI group and performed differential analysis on the basis of oedema classification, identifying 185 upregulated and 91 downregulated genes (Figure 1B). GSEA revealed significant activation of metabolic and inflammatory pathways—including ‘oxidative phosphorylation’, ‘glycolysis gluconeogenesis’, ‘Ecm receptor interaction’, ‘Nod‐like receptor signalling pathway’ and ‘Toll‐like receptor signalling pathway’ (Figure 1C).

### 3.3 Bulk and scRNA‐seq identified *Acp5* as a key signature gene associated with post‐TBI oedema

To explore oedema and inflammation associated transcriptional programs across species, we performed a cross‐context comparative analysis. We first analysed the transcriptional signature associated with human intracerebral haemorrhage (ICH) perihaematomal oedema using dataset GSE266873, in which brain oedema is a prominent secondary pathology. Subsequently, we compared these signals with the mouse TBI transcriptomic profiles from our bulk RNA‐seq data and the published scRNA‐seq dataset GSE175430, focusing on conserved pathways and cell‐type‐specific responses. GSE266873 data were classified into oligodendrocytes, astrocytes, neurons, endothelial cells, microglia, monocytes, neutrophils, NK/T cells and B cells (Figures 1D and S10). Moreover, the same gene set was used for AUCell, and cells were stratified into high‐ and low‐oedema score groups for differential analysis (Figure 1E). This approach resulted in the identification of 667 DEGs, comprising 362 upregulated and 305 downregulated genes. GSEA revealed significant activation of pathways such as ‘oxidative phosphorylation’, ‘glycolysis gluconeogenesis’, ‘complement and coagulation cascades’, ‘Alzheimer's disease’, ‘Ecm receptor interaction’ and ‘leukocyte transendothelial migration’ (Figure 1F). GSE175430 was subsequently subjected to Pearson correlation analysis to identify the top 100 genes that exhibited a strong correlation with brain oedema scores (Figure 1G). Differential analysis of the bulk RNA‐seq revealed that, compared with the sham group, 836 DEGs were identified at 1 day post‐TBI, comprising 762 upregulated and 74 downregulated genes. At 3 days post‐TBI, we observed a total of 1746 upregulated DEGs alongside 58 downregulated genes. By day 7 post‐TBI, a total of 1045 DEGs were identified, including 931 upregulated and 114 downregulated genes. Notably, the greatest number of DEGs was recorded on day 3 post‐TBI, underscoring its critical role in the overall progression of the disease. The intersection of the upregulated DEGs identified in GSE175430 with those highly correlated with brain oedema scores, along with the upregulated DEGs identified in GSE266873 and those identified as upregulated genes via bulk RNA‐seq at 1, 3 and 7 days post‐TBI compared with sham controls, revealed a total of nine key characteristic genes associated with brain oedema after TBI (Figure 1H and Table S3). GO enrichment analysis indicated a significant relationship with the regulation of inflammation and immunity. We identified several pathways closely linked to both brain oedema and neuroinflammation, including ‘regulation of immune effector processes’, ‘regulation of inflammatory responses’, ‘reactive oxygen species metabolic processes’, ‘tumour necrosis factor superfamily cytokine production’, and ‘myeloid leukocyte cytokine production’ (Figure 1I). Notably, all these pathways shared a common gene, *Acp5* (Figure 1J).

### 3.4 Single‐cell profiling identified ACP5 expression predominantly in monocytes/macrophages after TBI

**FIGURE 2:**
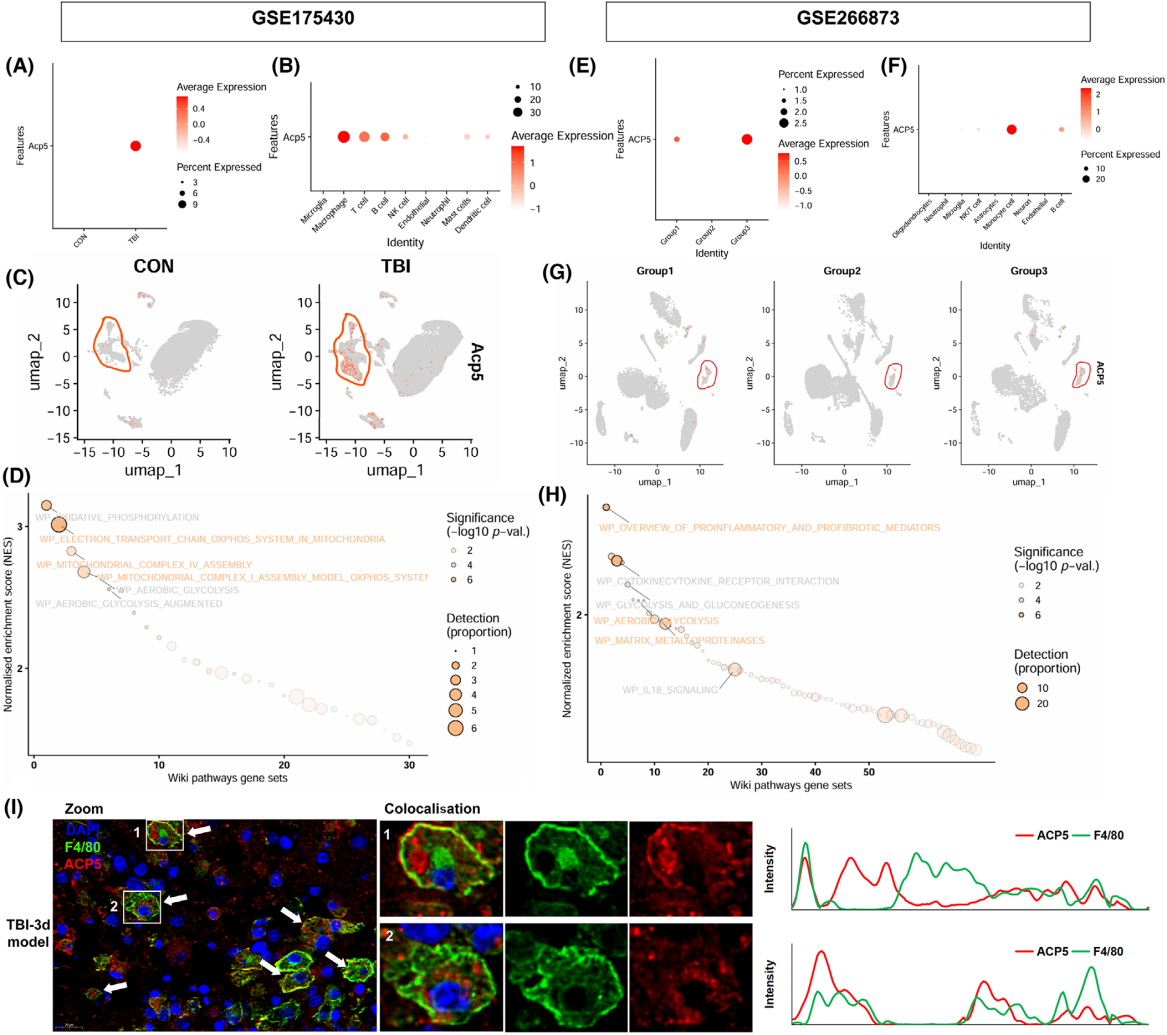
Single‐cell profiling identifies ACP5‐positive monocytes/macrophages associated with inflammatory programs in mouse traumatic brain injury (TBI) and human intracerebral haemorrhage (ICH). (A) Dot plots showing the expression of *Acp5* in different groups in GSE175430. (B) Dot plots showing the expression of *Acp5* in different cell types in GSE175430. (C) The uniform manifold approximation and projection (UMAP) plot specifically shows the expression distribution of *Acp5* in the control and TBI groups of the GSE175430 dataset, with monocytes/macrophages circled. (D) Gene set enrichment analysis (GSEA) revealed significantly enriched pathways in *Acp5*‐high macrophages in the GSE175430 dataset. (E) Dot plots showing the expression of *ACP5* in different groups in GSE266873. (F) Dot plots showing the expression of *ACP5* in different cell types in GSE266873. (G) The UMAP plot shows the distribution of *ACP5* expression across the three post‐ICH time‐window groups in GSE266873, with monocytes/macrophages highlighted. (H) GSEA revealed significantly enriched pathways in *ACP5*‐high macrophages in the GSE266873 dataset. (I) Immunofluorescence showing colocalisation of ACP5 (cytoplasm, red) with the peripheral macrophage marker F4/80 (membrane, green) in the brain lesion at 3 days post‐TBI. DAPI (blue) was used to stain the nucleus.

Single‐cell analyses identified ACP5‐enriched monocyte/macrophage populations across the mouse TBI and human ICH datasets. In the mouse GSE175430 dataset, *Acp5* expression was increased 3 days post‐injury and was predominantly detected in monocytes/macrophages (Figure 2A,B). In the human GSE266873 dataset, *ACP5* expression peaked in Group 3, corresponding to the 24–48 h post‐ICH interval and was similarly enriched in monocytes/macrophages (Figure 2E,F). UMAP visualisation further showed the distribution of ACP5‐positive cells within the monocyte/macrophage populations (Figure 2C,G). Differential analysis and GSEA revealed that ACP5‐high monocytes/macrophages exhibited enrichment of energy metabolism‐related pathways. These included enrichment in pathways such as ‘electron transport chain oxidative phosphorylation system in mitochondria’, ‘mitochondrial complex IV assembly’, and ‘overview of proinflammatory and profibrotic mediators’ (Figure 2D,H). Trajectory analysis further suggested that ACP5‐high cells are enriched during the terminal stages of monocyte/macrophage differentiation—a pattern conserved across species (Figures S13 and S14)—suggesting a potential association between ACP5‐positive cells and traumatic brain oedema that warrants further functional investigation. To assess their signalling potential, cell–cell interaction analyses predicted that ACP5‐high monocytes/macrophages presented the highest likelihood of both sending and receiving intense signals after TBI across murine and human datasets. Potential key pathways associated with these cells include proinflammatory cascades involving the SPP1, GALECTIN, MIF, VEGF and IL‐1 signalling pathways (Figures S15 and S16). Additionally, ligand‒receptor pairing analyses highlighted potential mechanisms for interactions between ACP5‐positive monocytes/macrophages and other cell types (Figures S17 and S18). Immunofluorescence showed colocalisation of ACP5 with F4/80‐positive macrophages (Figure 2I). These data reveal that ACP5‐positive monocytes/macrophages possess an inflammatory gene signature and active cell‐cell communication profile. Whether this population contributes to post‐TBI neuroinflammation requires direct functional testing.

### 3.5 ACP5 temporal expression dynamics, injury correlation and cellular localisation in TBI

**FIGURE 3:**
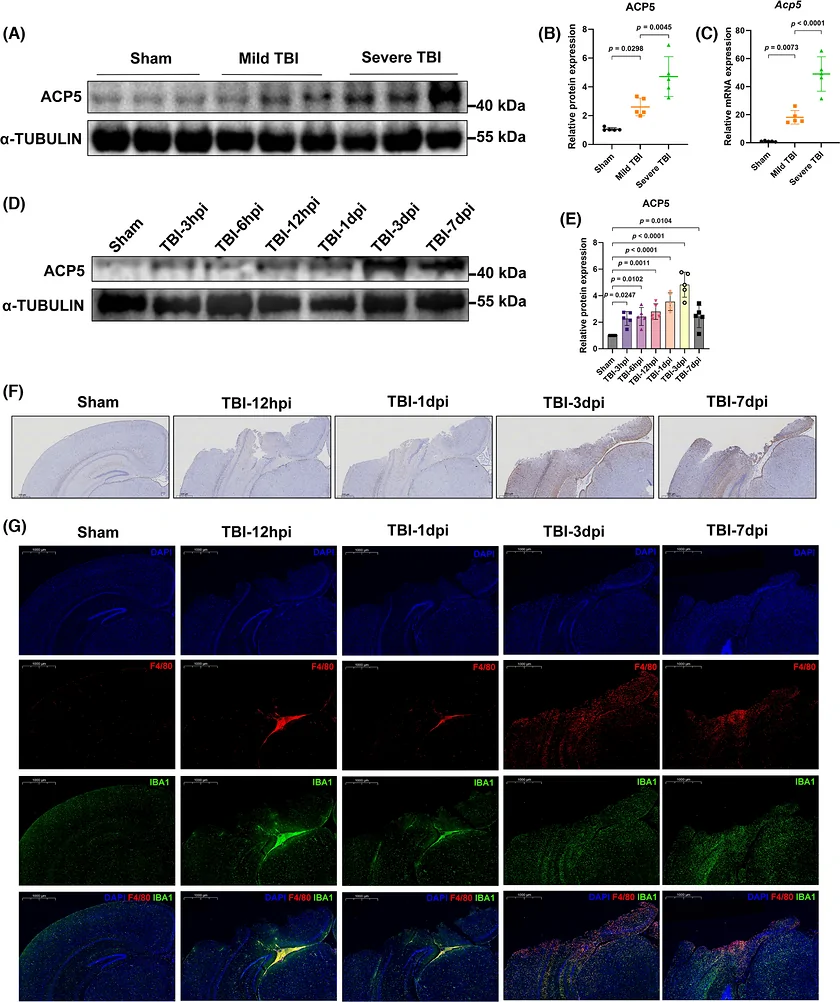
Temporal expression dynamics, injury correlation and cellular localisation of ACP5 in the traumatic brain injury (TBI) model. (A–C) Western blot (A, B) and reverse transcription quantitative polymerase chain reaction (RT‐qPCR) (C) showing ACP5 protein abundance and *Acp5* mRNA expression, respectively, at 3 days after mild and severe TBI. *N* = 5 per group. (D, E) Western blot analysis of ACP5 in brain tissue at various time points post‐TBI. *N* = 5 per group. (F) Immunohistochemical staining revealed the expression of ACP5 at various time points (12 h, 1 day, 3 days and 7 days) following TBI. (G) Immunofluorescence analysis of F4/80+ peripheral macrophage infiltration (red) and IBA1+ microglial distribution (green) at different time points post‐TBI. The data are expressed as the means ± standard deviations (SDs). Exact *p* values are shown in the figure. Values below  .0001 are reported as *p* < .0001.

To validate the findings obtained from the public database, we established a mouse model of TBI to assess the biological significance of ACP5 following TBI. We first evaluated whether *Acp5* is associated with the severity of injury. As shown in Figure 3A, *Acp5* expression levels were significantly positively correlated with TBI severity. Specifically, on day 3 after injury, both *Acp5* mRNA and ACP5 protein levels in brain tissue were significantly higher in mice with severe TBI compared to those with mild TBI (Figure 3B,C). Immunofluorescence demonstrated the infiltration of peripheral macrophages after mild and severe TBI (Figure S19). Subsequently, we examined the dynamic changes in *Acp5* expression after severe TBI. ACP5 expression gradually increased after injury, peaked at 3 days post‐TBI, and then declined progressively (Figure 3D,E), a pattern consistent with immunohistochemical staining results (Figure 3F). We utilised F4/80 to label peripheral infiltrating macrophages and IBA1 to label brain‐resident microglia as controls, allowing us to investigate their distribution at different time points post‐TBI. Notably, minimal infiltration of peripheral macrophages was detected in normal brain tissue, whereas microglia exhibited a typical scattered distribution. At 1 day post‐TBI, peripheral macrophage infiltration was minimal; however, by 3 days post‐injury, F4/80 expression reached its highest level in the injured area (Figure 3G). Additionally, microglia migrated towards this injury site after 3 days. Moreover, H&E staining revealed time‐dependent post‐TBI pathology, including widened perivascular spaces and a less densely stained parenchyma (Figure S20), changes commonly associated with oedema. Interestingly, *Acp5* expression in normal brain tissue was also notably low, suggesting a potential relationship with peripheral infiltrating macrophages.

### 3.6 ACP5 inhibition improved blood‒brain barrier integrity and neurological function after TBI

Before evaluating its effects in the TBI model, we characterised the concentration‐dependent pharmacological activity and cytotoxicity of AubipyOMe in RAW264.7 cells. Cell viability analysis showed no significant cytotoxicity at concentrations up to 10 µM, whereas higher concentrations reduced cell viability (Figure S25A). AubipyOMe inhibited ACP5 enzymatic activity in a concentration‐dependent manner, while its corresponding mRNA and protein abundance remained unchanged over the tested non‐cytotoxic concentration range (Figure S25B–D). These findings indicate that AubipyOMe acts primarily at the level of ACP5 enzymatic activity rather than by reducing its expression under the present in vitro conditions. We next performed an initial assessment of the systemic tolerability of the AubipyOMe regimen used in vivo. Complete blood count and serum biochemical analyses revealed no overt evidence of haematological, hepatic or renal toxicity following AubipyOMe administration, with all measured parameters remaining within the physiological reference ranges for mice (Figure S26A,B). Histological examination of the brain, heart, liver, spleen, lung and kidney revealed no overt treatment‐related pathological alterations (Figure S26C). Collectively, these findings provide preliminary evidence that the short‐term AubipyOMe regimen did not cause overt haematological or major‐organ toxicity at 28‐day assessment.

**FIGURE 4:**
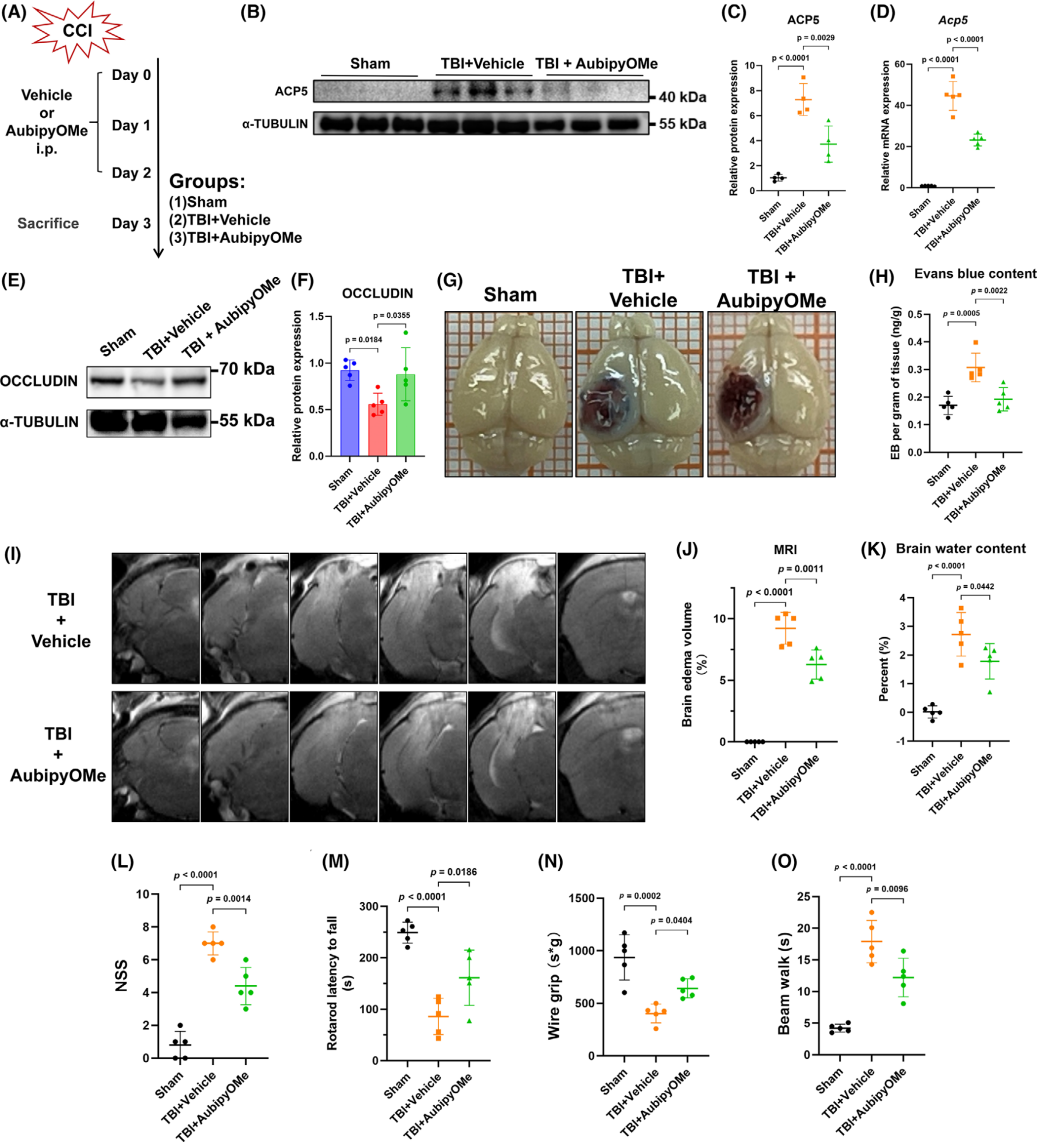
Pharmacological inhibition of ACP5 improves blood‒brain barrier integrity and neurological function after traumatic brain injury (TBI). (A) Flowchart of the application of AubipyOMe in animal experiments. (B–D) Western blot (B, C) and reverse transcription quantitative polymerase chain reaction (RT‐qPCR) (D) results showing reduced ACP5 protein abundance and *Acp5* mRNA expression, respectively, in perilesional brain tissue after AubipyOMe treatment. *N* = 5 per group. (E, F) Western blot (E) and quantification (F) of OCCLUDIN protein. *N* = 5 per group. (G, H) Representative brains (G) and quantification (H) of Evans blue extravasation. *N* = 5 per group. (I, J) Representative T2‐weighted magnetic resonance imaging (MRI) and quantification of cerebral oedema volume (J) showing a reduction in the AubipyOMe group. *N* = 5 per group. (K) Statistical analysis of the brain water content. *N* = 5 per group. (L‒O) Neurological and motor function tests, including the neurological severity score (NSS) (L), rotarod test (M), wire grip test (N) and balance beam test (O). *N* = 5 per group. The data are presented as the means ± SDs. Exact *p* values are shown in the figure. Values below  .0001 are reported as *p* < .0001.

To further elucidate the role of ACP5‐positive macrophages following TBI, we identified AubipyOMe, a highly effective small‐molecule inhibitor of ACP5 enzymatic activity, through a comprehensive literature review. Administration of AubipyOMe was accompanied by reduced *Acp5* mRNA and protein abundance in whole perilesional brain tissue at 3 days post‐TBI (Figure 4B–D). Notably, because AubipyOMe does not directly suppress *Acp5* expression in RAW264.7 macrophages, the observed in vivo decreases may partly reflect changes in the abundance or recruitment of ACP5‐positive cells rather than a direct transcriptional effect. To explore the function of this molecule thoroughly, we conducted a series of phenotypic experiments. OCCLUDIN is a tight junction protein whose expression is significantly inhibited after TBI but increases after AubipyOMe treatment (Figure 4E), which was confirmed by statistical analysis (Figure 4F), indicating that the integrity of the BBB has improved. Additionally, an Evans blue experiment confirmed that dye leakage into the brain parenchyma was markedly lower in the AubipyOMe group than in the vehicle group (Figure 4G,H). We also employed MRI to assess the extent of brain injury and oedema following TBI (Figure 4I). Cerebral oedema volume was reduced in AubipyOMe‐treated mice (Figure 4J). The water content in the injured hemisphere of brain tissue from mice treated with AubipyOMe was significantly lower than that in the vehicle group (Figure 4K). Furthermore, we evaluated neurological and motor functions via short‐term behavioural assessments. The NSS (Figure 4L), rotarod test (Figure 4M), wire grip test (Figure 4N) and balance beam test (Figure 4O) results revealed significant improvements in neurological function after treatment with AubipyOMe post‐TBI.

### 3.7 ACP5 inhibition reduced infiltrating macrophage accumulation in brain tissue at 3 days post‐TBI

**FIGURE 5:**
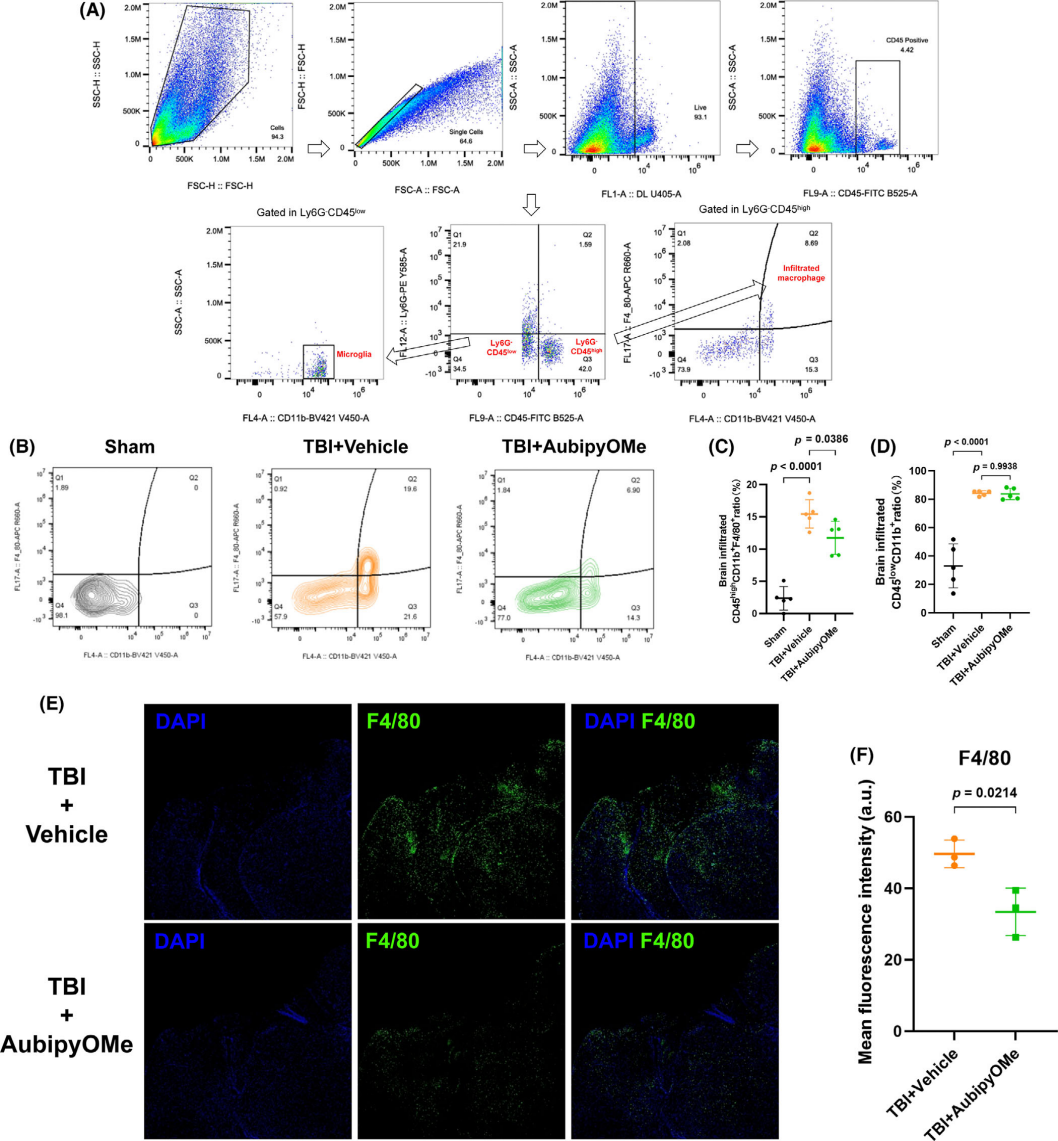
ACP5 inhibition reduces infiltrating macrophage accumulation in brain tissue at 3 days post‐traumatic brain injury (TBI). (A) Gating strategy for flow cytometry analysis. (B, C) (B) Representative flow cytometry plots and quantification (C) showing the decreased percentage of peripheral infiltrating macrophages (CD45^high^CD11b + F4/80+) upon AubipyOMe treatment. (D) Quantification of microglia (CD45^low^CD11b+) revealed no significant change after AubipyOMe treatment. *N* = 5 per group. (E, F) Representative immunofluorescence images (E) and quantification of the fluorescence intensity (F) of F4/80 (green) in the lesion area. *N* = 3 per group. The data are presented as the means ± SDs. Exact *p* values are shown in the figure. Values below  .0001 are reported as *p* < .0001.

In addition to validating functional phenotypes, we further investigated the underlying mechanism via flow cytometry and immunofluorescence techniques. The flow cytometry gating strategy was as follows (Figure 5A): (1) single cells were gated, and live cells were identified through viability staining; (2) CD45, a panleukocyte marker, was employed to isolate leukocytes; (3) Ly6G was utilised to exclude contamination from multinucleated cells such as neutrophils; and (4) CD11b‐positive cells within the CD45^low^ population (CD45^low^CD11b+) were classified as microglia, whereas double‐positive CD11b and F4/80 cells in the CD45^high^ population (CD45^high^CD11b + F4/80+) were designated peripheral infiltrating macrophages. The results indicated that peripheral infiltrating macrophages exhibited substantial infiltration post‐TBI, which was consistent with previous findings, and peripheral macrophage infiltration decreased upon inhibition of ACP5 (Figure 5B,C). Additionally, there was a significant increase in microglial infiltration; however, no notable changes occurred after the administration of AubipyOMe (Figure 5D). Immunofluorescence analysis further revealed that the fluorescence intensity of F4/80 was significantly lower in the AubipyOMe group than in the control group (Figure 5E,F).

### 3.8 ACP5 perturbation is associated with CSF2‐related JAK/STAT and NF‐κB signalling in vivo and in vitro

**FIGURE 6:**
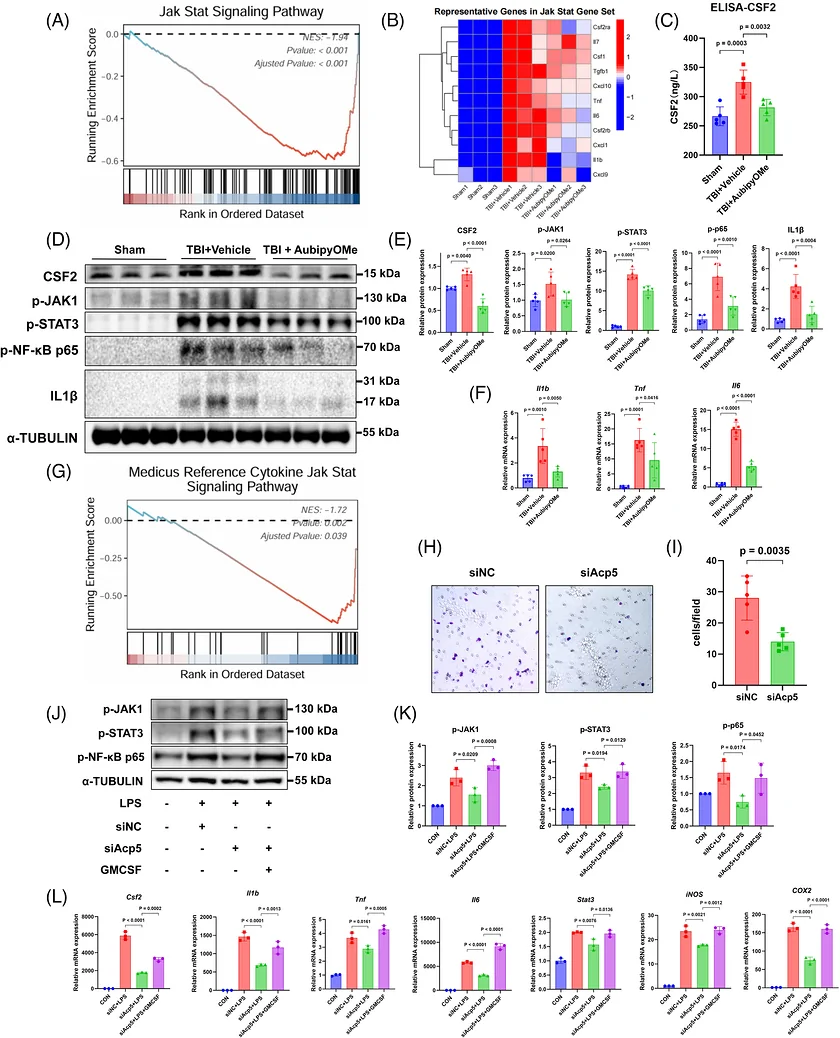
ACP5 perturbation is associated with CSF2‐related JAK/STAT and NF‐κB signalling in vivo and in vitro. (A) Gene set enrichment analysis (GSEA) plot showing negative enrichment of the JAK–STAT signalling pathway in brain tissue from AubipyOMe‐treated mice compared with vehicle‐treated traumatic brain injury (TBI) mice. (B) Heatmap showing representative cytokines and signalling‐related genes associated with the JAK–STAT pathway. (C) Enzyme‐linked immunosorbent assay (ELISA) analysis of the CSF2 concentration in the sham, TBI + Vehicle and TBI + AubipyOMe groups. (D, E) Western blot (D) and quantification (E) showing the protein levels of CSF2, p‐JAK1, p‐STAT3, p‐NF‐κB p65 and IL‐1β. (F) Reverse transcription quantitative polymerase chain reaction (RT‐qPCR) results showing the mRNA levels of the proinflammatory factors. (G) GSEA plot showing negative enrichment of the JAK–STAT signalling pathway following *Acp5* knockdown in lipopolysaccharide (LPS)‐stimulated RAW264.7 cells. (H, I) Transwell assay showing the effects of *Acp5* knockdown on the migration of RAW264.7 cells. (J, K) The results of western blot (J) and quantitative analysis (K) indicated that si*Acp5* transfection downregulated the expression of p‐JAK1, p‐STAT3 and p‐NF‐κB p65 in RAW264.7 cells stimulated with LPS, whereas exogenous supplementation with CSF2 reversed the downregulatory effect of si*Acp5* on p‐JAK1, p‐STAT3 and p‐NF‐κB p65. (L) RT‐qPCR of *Csf2*, *Il1b*, *Tnf*, *Il6*, *Nos2* (iNOS), *Ptgs2* (COX‐2) and *Stat3* in RAW264.7 cells followed by si*Acp5* transfection and exogenous supplementation with CSF2. *N* = 5 per group for animal experiments; *N* = 3 independent biological replicates per group for cell experiments. The data are presented as the means ± SDs. Exact *p* values are shown in the figure. Values below  .0001 are reported as *p* < .0001.

Given the significant role of neuroinflammation in the occurrence and development of brain oedema, we explored the relationship between ACP5 and inflammatory factors through RNA sequencing and molecular biology experiments. Compared with those in the vehicle group, the ‘Toll‐like receptor signalling pathways’, ‘Nod‐like receptor signalling pathways’, ‘Cytosolic DNA sensing pathways’, ‘Cell adhesion molecule Cams’, ‘Leukocyte transendothelial migration’ and ‘ECM receptor interaction’ pathways were negatively enriched in the AubipyOMe group (Figure S21), suggesting that AubipyOMe treatment comprehensively improved the inflammatory environment in brain tissue after TBI, providing some insights into the role of ACP5. Immune infiltration analysis indicated that after AubipyOMe treatment, the inferred abundance of dendritic cells, CD4+ T cells and macrophages was reduced (Figure S22). GSEA indicated attenuation of JAK–STAT‐related transcriptional programs following AubipyOMe treatment (Figure 6A). We subsequently selected representative cytokines and related genes associated with this pathway,^48^ and the resulting heatmap illustrated their change patterns (Figure 6B). A literature review indicated that CSF2 is an upstream activator of JAK–STAT signalling.^49^ ELISA showed that the CSF2 concentration in perilesional brain tissue was reduced after the administration of AubipyOMe (Figure 6C), which is in line with our hypothesis. Western blot analysis confirmed that the protein expression levels of CSF2, p‐JAK1, p‐STAT3, p‐NF‐κB p65 and IL‐1β in brain tissue were significantly reduced following inhibitor treatment (Figure 6D,E). RT‐qPCR was used to assess the expression of classical proinflammatory factors, revealing a substantial decrease in their levels (Figure 6F). To further elucidate the role of ACP5, we utilised siRNA to knockdown *Acp5* in RAW264.7 cells and validated the knockdown efficiency through western blot and RT‐qPCR (Figure S23). LPS was subsequently used to induce an inflammatory response after transfection, followed by RNA‐seq analysis. The results demonstrated that overall inflammatory pathways in RAW264.7 cells were significantly inhibited after si*Acp5* (Figure S24), such as ‘Overview of proinflammatory and profibrotic mediators’, ‘Inflammatory response pathway’, ‘Cytokines and inflammatory response’, ‘Cytokine‒cytokine receptor interaction’ and ‘Il18 signalling’; notably, ‘Cytokine jak stat signalling pathways’ remained markedly suppressed (Figure 6G). The transwell assay results demonstrated that *Acp5* knockdown significantly inhibited the migration of macrophages (Figure 6H,I). In RAW264.7 cells, *Acp5* knockdown via siRNA suppressed the inflammatory response, as evidenced by decreased protein levels of p‐JAK1, p‐STAT3 and p‐NF‐κB p65 (Figure 6J,K) and reduced transcription of inflammatory mediators (Figure 6L). Importantly, exogenous CSF2 partially restored downstream signalling and inflammatory‐gene expression after *Acp5* knockdown, supporting CSF2 as a candidate mediator of the ACP5‐associated inflammatory response in this model; these experiments do not establish a direct molecular link between ACP5 and *Csf2* transcription.

### 3.9 Global *Acp5* deletion attenuates acute inflammatory phenotypes and improves long‐term behavioural outcomes after TBI

**FIGURE 7:**
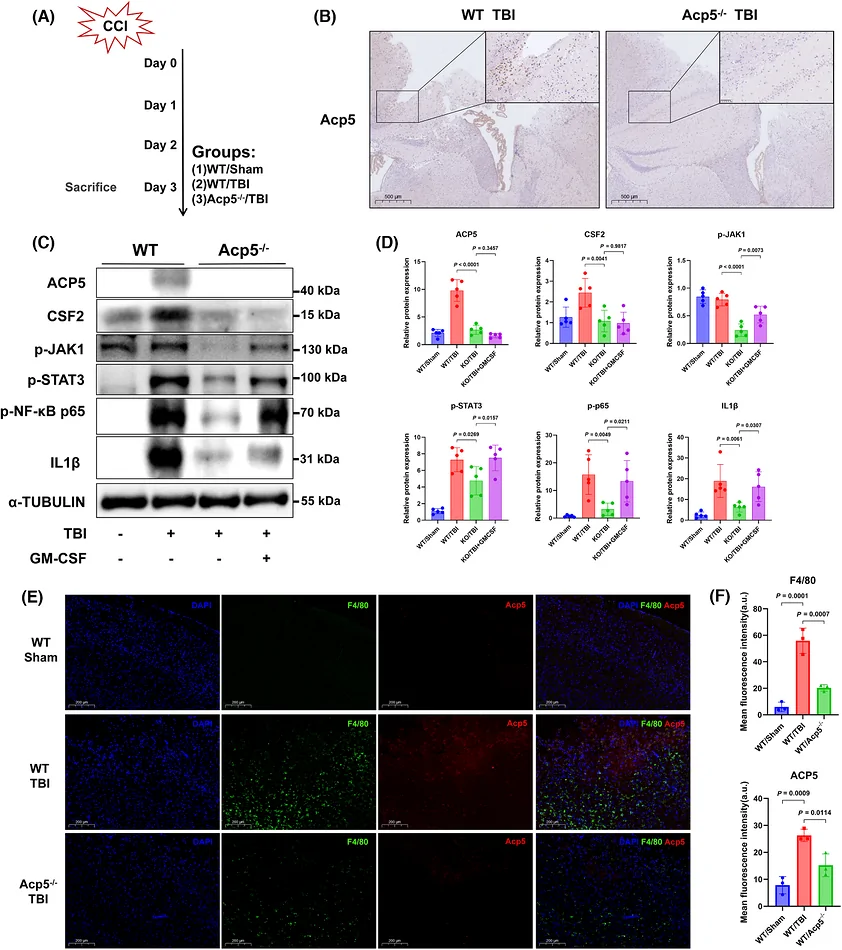
Global *Acp5* deletion attenuates inflammatory signalling and macrophage accumulation after traumatic brain injury (TBI). (A) Flowchart of the experimental procedure for *Acp5*‐KO mice. (B) Immunohistochemistry (IHC) staining confirmed loss of ACP5 protein expression in *Acp5*‐KO mice. (C, D) Western blot (C) and quantification (D) of the protein levels of ACP5, CSF2, p‐JAK1, p‐STAT3, p‐NF‐κB p65 and IL‐1β in the wild‐type (WT) sham, WT TBI, *Acp5*‐KO TBI and *Acp5*‐KO TBI + CSF2 groups at 3 days post‐injury. *N* = 5 per group. (E, F) Representative immunofluorescence images (E) and quantification of the fluorescence intensity (F) of F4/80 (green) and ACP5 (red) in the lesion area. *N* = 3 per group. The data are presented as the means ± SDs. Exact *p* values are shown in the figure. Values below  .0001 are reported as *p* < .0001.

To further assess the contribution of ACP5 to TBI‐associated phenotypes, we used global *Acp5‐*KO mice. Immunohistochemistry (IHC) and western blotting confirmed efficient loss of ACP5 protein compared with their WT littermates (Figure 7B). Following TBI, we assessed the levels of key inflammatory mediators at 3 days post‐injury. Western blot analysis revealed significant decreases in the expression of CSF2, p‐JAK1, p‐STAT3, p‐NF‐κB p65 and IL‐1β in the perilesional brain tissue of *Acp5*‐KO mice compared with that in the TBI WT controls (Figure 7C,D). To further determine whether CSF2 functionally mediates the inflammatory changes associated with *Acp5* deletion in vivo, we performed a CSF2 rescue experiment in *Acp5*‐KO mice following TBI. Compared with the *Acp5*‐KO TBI group, CSF2 administration partially restored the levels of p‐JAK1, p‐STAT3, p‐NF‐κB p65 and IL‐1β in perilesional brain tissue at 3 days post‐injury (Figure 7C,D). These findings support CSF2 as a functional downstream mediator of ACP5‐associated inflammatory signalling in vivo. Consistent with the attenuation of inflammatory signalling, immunofluorescence analysis revealed reduced F4/80‐positive macrophage accumulation in the injured hemisphere of *Acp5*‐KO mice compared with WT mice at 3 days post‐TBI (Figure 7E,F).

**FIGURE 8:**
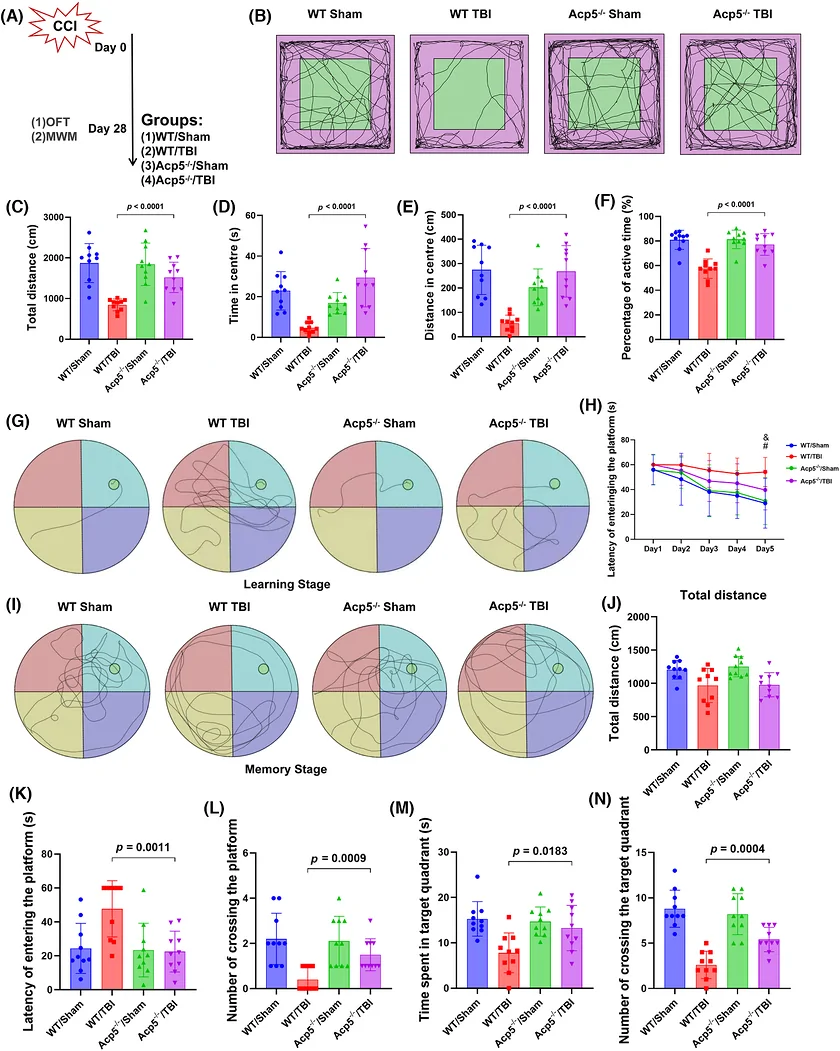
Global *Acp5* deletion improves long‐term behavioural outcomes after TBI. (A) Flowchart of the experimental procedure for *Acp5*‐KO mice. (B) Representative images of the moving routes of the mice in the open field test. (C–F) Quantitative analysis of total distance travelled (C), time spent in the centre (D), distance travelled in the centre (E) and percentage of active time (F) in the open field test. (G) Representative images of the swimming routes of the learning stage in the Morris water maze. (H) Latency to find the platform in the learning stage (&, *p* = .0103, wild‐type (WT)/TBI vs. WT/Sham; #, *p* = .0829, WT/TBI vs. *Acp5*‐KO/TBI). (I) Representative images of the swimming routes of the memory stage in the Morris water maze. (J–N) Quantitative analysis of the total distance travelled (J), latency of platform entry (K), number of platform crossings (L), time spent in the target quadrant (M) and number of entries into target quadrants (N) in the probe trial. *N* = 10 per group. The data are presented as the means ± SDs. Exact *p* values are shown in the figure. Values below  .0001 are reported as *p* < .0001.

To evaluate the long‐term functional consequences of *Acp5* deletion, we performed behavioural tests to assess cognitive function at 28 days post‐TBI (Figure 8A). Open field testing was used to assess locomotor activity and anxiety‐like behaviour. In the open field test, *Acp5*‐KO mice subjected to TBI exhibited significantly greater performance than did TBI WT mice across multiple parameters (Figure 8B): total distance travelled (Figure 8C), time spent in the centre zone (Figure 8D), distance travelled in the centre (Figure 8E), and the ratio of active time (Figure 8F). Notably, under baseline sham conditions, no significant differences in these metrics were detected between *Acp5*‐KO and WT mice, indicating that *Acp5* deletion does not inherently alter general locomotor activity or anxiety‐like behaviour. Spatial learning and memory were assessed via the MWM. During the 5‐day acquisition phase, *Acp5*‐KO mice displayed a trend towards shorter escape latencies than WT TBI mice did (Figure 8G,H). In the probe trial conducted 24 h after the final training session, both TBI groups exhibited reduced swimming speed (Figure S27) and total swimming distance (Figure 8J) compared with their corresponding sham groups. Swimming speed did not differ significantly between the WT‐TBI and *Acp5*‐KO‐TBI groups, suggesting that genotype‐dependent differences in swimming ability were unlikely to account for their different water‐maze performance. Under these conditions, *Acp5*‐KO‐TBI mice showed better memory retention than WT‐TBI mice (Figure 8I,K–N). This was evidenced by a shorter latency to first enter the platform location (Figure 8K), a greater number of platform crossings (Figure 8L), increased time spent in the target quadrant (Figure 8M) and a greater number of entries into the target quadrant (Figure 8N) than in WT TBI mice. However, no significant differences in water‐maze performance were detected between sham‐operated WT and *Acp5*‐KO mice, suggesting that the genotype did not produce an evident baseline difference under sham conditions. Collectively, these genetic findings provide orthogonal evidence that global *Acp5* deletion is associated with attenuated acute inflammatory responses and improved long‐term behavioural outcomes after TBI.

## 4 DISCUSSION

In this study, we investigated peripheral ACP5‐positive monocytes/macrophages in experimental TBI and found that their accumulation was associated with cerebral oedema and neurological dysfunction. Pharmacological inhibition with AubipyOMe was associated with improved BBB integrity, reduced brain oedema and improved neurological function at 3 days post‐injury. In addition, ACP5 inhibition was accompanied by reduced expression of proinflammatory cytokines, including IL‐1β, TNF‐α and IL‐6, together with attenuation of CSF2‐related JAK/STAT and NF‐κB signalling. Although the direct molecular relationship between ACP5 and this signalling axis remains to be clarified, these findings support ACP5 as a preclinical candidate for further investigation in secondary TBI injury.

Cerebral oedema is a life‐threatening complication following TBI and presents a significant clinical challenge in neurocritical care.^50^, ^51^ According to the Monro‐Kellie doctrine, an increase in brain volume due to cerebral oedema leads to a rapid elevation in the ICP.^52^ Elevated ICP has been identified as a predictor of poor prognosis in patients with TBI, stroke and other intracranial lesions.^53^ One study indicated that approximately half of the patients experienced their highest average ICP within the first 3 days post‐injury; however, 25% exhibited delayed increases in ICP after the fifth day.^54^ Although standard treatments, including osmotherapy, sedation and surgical intervention, are currently widely employed, these approaches appear to have little effect on neurological outcomes.^53^, ^55^ With the deepening understanding of brain oedema after TBI, the latest advancements in preclinical models of TBI have revealed potential molecular targets that may alleviate brain oedema and its adverse consequences.^56^, ^57^, ^58^, ^59^, ^60^ However, the heterogeneity of TBI‐related injuries and the complex inflammatory cascade mechanisms leading to brain oedema pose significant challenges.^61^ Targeted treatments for neuroinflammation‐driven brain oedema remain a key challenge in this research field,^62^ highlighting the urgent need for innovative therapeutic strategies.

Given the multifactorial nature of post‐TBI cerebral oedema, the oedema‐associated score used in this study should be interpreted as a composite molecular proxy rather than a direct measurement of tissue water content. The 97‐gene signature was predominantly enriched in processes related to BBB integrity, vascular permeability, vascular regulation and tissue homeostasis, which are central components of cerebral oedema pathophysiology. In addition, the signature contains genes directly implicated in water transport and ion homeostasis, including *Aqp4*, *Aqp9*, *Slc12a2*, *Atp1a2*, *Trpm4* and *Trpv4*. Nevertheless, neuroinflammation, vascular injury and oedema are biologically interconnected after TBI. Therefore, the score is not intended to be completely independent of inflammatory activation; rather, it captures oedema‐relevant vascular, BBB and water/ion‐homeostasis‐related components within this interconnected pathological context. Its correlation with MRI‐derived oedema volume supports the biological relevance of this composite molecular score (Figure S7).

Within this pathophysiological framework, tartrate‐resistant acid phosphatase 5 (TRAP/ACP5; encoded by *ACP5*) is a relatively conserved and multifunctional protein^63^ located at the 19p13.2–p13.3 locus on human chromosome 19 and can be involved in the generation of reactive oxygen species and the regulation of macrophage function.^64^ Studies have demonstrated that ACP5 is expressed predominantly in myeloid cells,^65^, ^66^ including granulocytes and mononuclear phagocytes, with particular emphasis on its role as a marker for osteoclasts.^67^ The activity and function of ACP5 may vary across different cell types.^63^ While ACP5 is physiologically expressed, its elevated levels can be observed in various pathological conditions, such as malignant tumours,^68^, ^69^ metabolic disorders such as Gaucher's disease,^70^ and abnormalities in bone development.^71^ These findings suggest that ACP5 has extensive pathophysiological functions. However, the role of ACP5 within the nervous system remains largely underexplored. Therefore, on the basis of the observed correlation between ACP5 and both the time course and severity following TBI, we hypothesise that ACP5 plays a significant role in progression after TBI. This study is the first to report that ACP5 may be involved in secondary injury subsequent to TBI.

CNS‐resident microglia and astrocytes are traditionally regarded as the primary mediators of post‐TBI neuroinflammatory responses.^72^ Although systemic circulating immune cells and inflammatory molecules play crucial roles in TBI,^73^ research in this area remains limited. Studies have shown that macrophages contribute to pathogenesis during the chronic phase following TBI.^74^, ^75^ Monocyte‐derived macrophages reach their peak numbers 24–48 h after brain injury in both animal models and humans^19^; however, their involvement in the acute phase of TBI pathogenesis is still not fully understood. Our identification of ACP5‐positive monocytes/macrophages as contributors to cerebral oedema and neurological dysfunction is consistent with emerging evidence that highlights the involvement of peripheral immune cells in neuroinflammation.^12^, ^76^ Given that the infiltration time window of ACP5‐positive macrophages (1–3 days) aligns closely with disease progression, elucidating the mechanisms underlying this significant phenomenon may offer a promising new therapeutic strategy for mitigating secondary injury after TBI.

Granulocyte‐macrophage colony‐stimulating factor (GM‐CSF/CSF2), encoded by the *Csf2* gene, is emerging as a crucial intermediary linking tissue‒invasive myeloid cells across various disease contexts.^77^, ^78^, ^79^ This factor is produced by multiple cell types, including both myeloid cells and lymphocytes.^79^ Recent studies have shown that CSF2 is a regulator of myeloid cell activation under inflammatory conditions and has evolved into a key proinflammatory cytokine that participates in the pathogenesis of various inflammatory and autoimmune diseases.^80^, ^81^ Blocking CSF2 significantly reduces joint swelling and cartilage damage in mouse models of chronic arthritis.^82^ CSF2‐induced inflammatory phagocyte infiltration predominantly occurs in the CNS, liver and lungs. Compared with inflammatory phagocytes found in other organs, those infiltrating the CNS produce reactive oxygen species and exhibit distinct genetic characteristics.^78^ This observation highlights a potential avenue for specifically targeting myeloid cells within the CNS. Our study revealed that CSF2 is significantly upregulated in both animal models of TBI and cellular models of neuroinflammation. Notably, further mechanistic investigations revealed that *Acp5* knockdown reduced LPS‐induced CSF2 production as well as the subsequent secretion of proinflammatory cytokines. These findings suggest that ACP5 may influence inflammatory responses partly through changes in CSF2 expression and downstream signalling. The binding of CSF2 to its receptor activates JAK–STAT signalling, along with other downstream pathways, such as the MAPK, PI3K–AKT and NF‐κB pathways.^79^, ^83^ Collectively, these findings identify ACP5‐positive monocytes/macrophages as an injury‐associated myeloid population that may contribute to post‐TBI neuroinflammation. Our findings suggest that CSF2 may act as a downstream mediator of ACP5‐associated inflammatory signalling. However, the direct molecular relationship between ACP5 and CSF2 regulation, as well as the functional intercellular communication predicted by the computational analyses, requires further experimental investigation. These findings support the potential involvement of ACP5‐positive macrophages and CSF2‐related signalling in post‐TBI neuroinflammation and provide a basis for further mechanistic studies evaluating this pathway as a candidate for therapeutic intervention.

Our study has several limitations. First, because suitable human TBI single‐cell datasets containing sufficient brain‐infiltrating myeloid cells were unavailable, the human analysis relied on an ICH dataset as a cross‐condition comparator. Although TBI and ICH share features of cerebral oedema and neuroinflammation, their pathophysiological mechanisms differ; therefore, these findings cannot be considered direct validation in human TBI. Furthermore, ACP5 has not been evaluated in human TBI tissue, blood, cerebrospinal fluid or imaging‐linked clinical cohorts, and its diagnostic or prognostic value remains unknown. Second, the oedema‐associated transcriptional score represents a composite molecular proxy rather than a direct measurement of tissue water content. Although its correlation with MRI‐derived oedema volume supports its biological relevance, validation in larger independent cohorts using complementary measures of oedema is warranted. Third, the global *Acp5*‐KO model cannot distinguish the contribution of infiltrating macrophages from systemic or non–cell‐autonomous effects. Lineage‐restricted deletion, fate mapping or bone‐marrow‐chimera studies will therefore be required to establish the cell‐specific role of ACP5. Fourth, although the in vitro and in vivo CSF2‐rescue experiments support CSF2 as a functional downstream mediator of the ACP5‐associated inflammatory phenotype, the direct molecular mechanism linking ACP5 to CSF2 regulation remains unresolved. Future studies using selective pathway inhibitors and genetic perturbation will be required to define the causal signalling hierarchy. Fifth, validation in larger cohorts remains desirable, and the exclusive use of male mice limits the generalisability of the findings across sexes. Finally, spatially resolved and longitudinal studies will be needed to define the anatomical distribution, heterogeneity and chronic functions of ACP5‐positive myeloid cells after TBI.

## 5 CONCLUSION

In conclusion, our findings identify ACP5‐positive macrophages as an injury‐associated myeloid population contributing to secondary injury after experimental TBI. Complementary pharmacological and genetic evidence supports the involvement of ACP5 and CSF2‐related inflammatory signalling in this process. These findings provide preclinical evidence supporting further investigation of ACP5 in TBI.

## AUTHOR CONTRIBUTIONS

All the authors had full access to the data in the study and take responsibility for the integrity of the data and the accuracy of the data analysis. Conceptualisation, Zhang Yulian and Yu Yanbing; Methodology, Dang Hanhan, He Kun and Zhang Yulian; Investigation, Zhang Yunsheng, Wang Zixi, Yang Xu and Chen Pengyu; Formal analysis, Dang Hanhan and Zhang Chuanpeng; Writing — original draft, Dang Hanhan; Writing — review and editing, Dang Hanhan, He Kun and Zhang Yulian; Visualisation, Dang Hanhan and He Kun; Funding acquisition, Zhang Yulian and Yu Yanbing; Supervision and project administration, Zhang Li and Yu Yanbing.

## CONFLICT OF INTEREST STATEMENT

The authors declare no conflicts of interest.

## ETHICS STATEMENT

All animal experiments were approved by the Institutional Animal Experimental Committee (approval No. ZRDWLL240035).

## Supporting information



Supporting Information File 01: ctm270845‐sup‐0001‐SuppMat.pdf

## Data Availability

The single‐cell RNA‐sequencing datasets are publicly available in GEO under accession numbers GSE175430 and GSE266873. The bulk RNA‐sequencing count matrix underlying the TBI time‐course analysis is available in Zenodo at https://doi.org/10.5281/zenodo.22544463. The complete 97‐gene oedema‐associated signature is provided in Table S2. All other data are available from the corresponding author upon reasonable request.
