# Supplementary material for: ACP5‐positive macrophages contribute to cerebral oedema and neuroinflammation after traumatic brain injury
Source: Clin Transl Med. 2026 Sep 25;16(9):e70845. doi: 10.1002/ctm2.70845 (PMC13613102; doi:10.1002/ctm2.70845)

Supplemental Materials

Table S1. Primer sequences used for RT-qPCR.

Name	Primer sequence (5′–3′)
Acp5	F: 5’ GCTGTCCTGGCTCAAAAAGC 3’
	R: 5’ GTAGGCAGTGACCCCGTATG 3’
Tnf	F: 5’ GATCGGTCCCCAAAGGGATG 3’
	R: 5’ CCACTTGGTGGTTTGTGAGTG 3’
Il1b	F: 5’ TGTAATGAAAGACGGCACACC 3’
	R: 5’ TCTTCTTTGGGTATTGCTTGG 3’
Il6	F: 5’ GCTACCAAACTGGATATAATCAGGA 3’
	R: 5’ CCAGGTAGCTATGGTACTCCAGAA 3’
Csf2	F: 5’ CATGCCTGTCACGTTGAATGA 3’
	R: 5’ AAATTGCCCCGTAGACCCTG 3’
Nos2 (iNOS)	F: 5’ GTTCTCAGCCCAACAATACAAGA 3’
	R: 5’ GTGGACGGGTCGATGTCAC 3’
Ptgs2 (COX-2)	F: 5’ GCTCAGCCAGGCAGCAAATC 3’
	R: 5’ ACCATAGAATCCAGTCCGGGT 3’
Stat3	F: 5’ AGGACATCAGTGGCAAGACC 3’
	R: 5’ CCTTGGGAATGTCGGGGTAG 3’
Gapdh	F: 5’ AACTTTGGCATTGTGGAAGG 3’
	R: 5’ GGATGCAGGGATGATGTTCT 3’

Table S2. Final 97-gene cerebral edema-associated signature derived from GeneCards candidates and Mfuzz clustering of bulk RNA-seq time-course data.

Cerebral Edema Genes from GeneCards							
Cluster1(n=67)				Cluster2(n=97)/Final Gene Set			
Abcc8	Fgf2	Ndufs4	Slc12a1	Abcb4	Dguok	Mip	Slc1a3
Acad9	Glul	Nt5e	Slc1a2	Acadm	Echs1	Mmp2	Slc22a5
Ager	Gpt	Odc1	Slc25a12	Acads	Egf	Mmp9	Slc25a10
Alb	Hccs	Pah	Slc25a22	Acadvl	Egfr	Mpo	Slc25a13
Angpt2	Ido1	Panx1	Slc25a3	Afp	Esam	Mpv17	Slc25a15
Aqp1	Il1b	Pex1	Sms	Ahcyl	Etfa	Mto1	Slc25a20
Astn2	Il1rn	Plat	Sod2	Alox5	Etfb	Myd88	Slc38a5
Avp	Il6	Pnkd	Src	Angpt1	Etfdh	Naxd	Slc4a4
Bdkrb2	Jun	Prkca	Tac1	Anxa1	F3	Naxe	Slc7a11
Cacna1a	Kcna1	Prkcb	Tbk1	Apoe	Fasl	Nes	Sod1
Cacna1b	Kcnq2	Ranbp2	Tfrc	Aqp4	Fgfr1	Nf2	Stat3
Cbs	Klb	Scn1a	Tgfb2	Aqp9	Gfap	Nlrp3	Tgfb1
Coq6	Lias	Serac1	Tmem126b	Arg1	Gja1	Nos2	Timp2
Cps1	Mapk1	Serpine1	Tnf	Asl	Gsr	Nos3	Tjp1
Edn1	Mir23b	Setd2	Vegfa	Ass1	Hadha	Notch3	Tjp2
Epor	Myc	Shh	Wnk3	Atp1a2	Hif1a	Nrp1	Tlr3
Fgf14	Ndufaf1	Sirt1		Bax	Hmgcl	Ocln	Tlr4
				Bckdha	Hmox1	Parp1	Tnc
				Bcs1l	Icam1	Pik3c2a	Trpm4
				Casp8	Idh1	Prrt2	Trpv4
				Cd274	Kcnn4	Ptn	Tspo
				Cldn5	Kdr	Rgma	Vim
				Col4a2	Lama2	S100b	
				Cpt2	Mapk3	Slc12a2	
				Ctnnb1	Mgmt	Slc19a3	

Table S3. List of 9 intersection genes identified from the multi-omics screening (related to Figure 1H).

Gene Symbol	Gene Name
Acp5	Acid phosphatase 5
Spp1	Secreted phosphoprotein 1 (osteopontin)
Vim	Vimentin
Hmox1	Heme Oxygenase-1
Thbs1	Thrombospondin-1
Lgals1	Galectin-1
Plin2	Perilipin-2
Lgals3	Galectin-3
F13a1	Coagulation factor XIII A chain

Figure S1. Venn diagram illustrating the results obtained from various search strategies. The red section denotes the 211 common intersection genes. BE: "brain edema"; CE: "cerebral edema"; TBIBE: "traumatic brain injury" and "brain edema"; TBICE: "traumatic brain injury" and "cerebral edema".

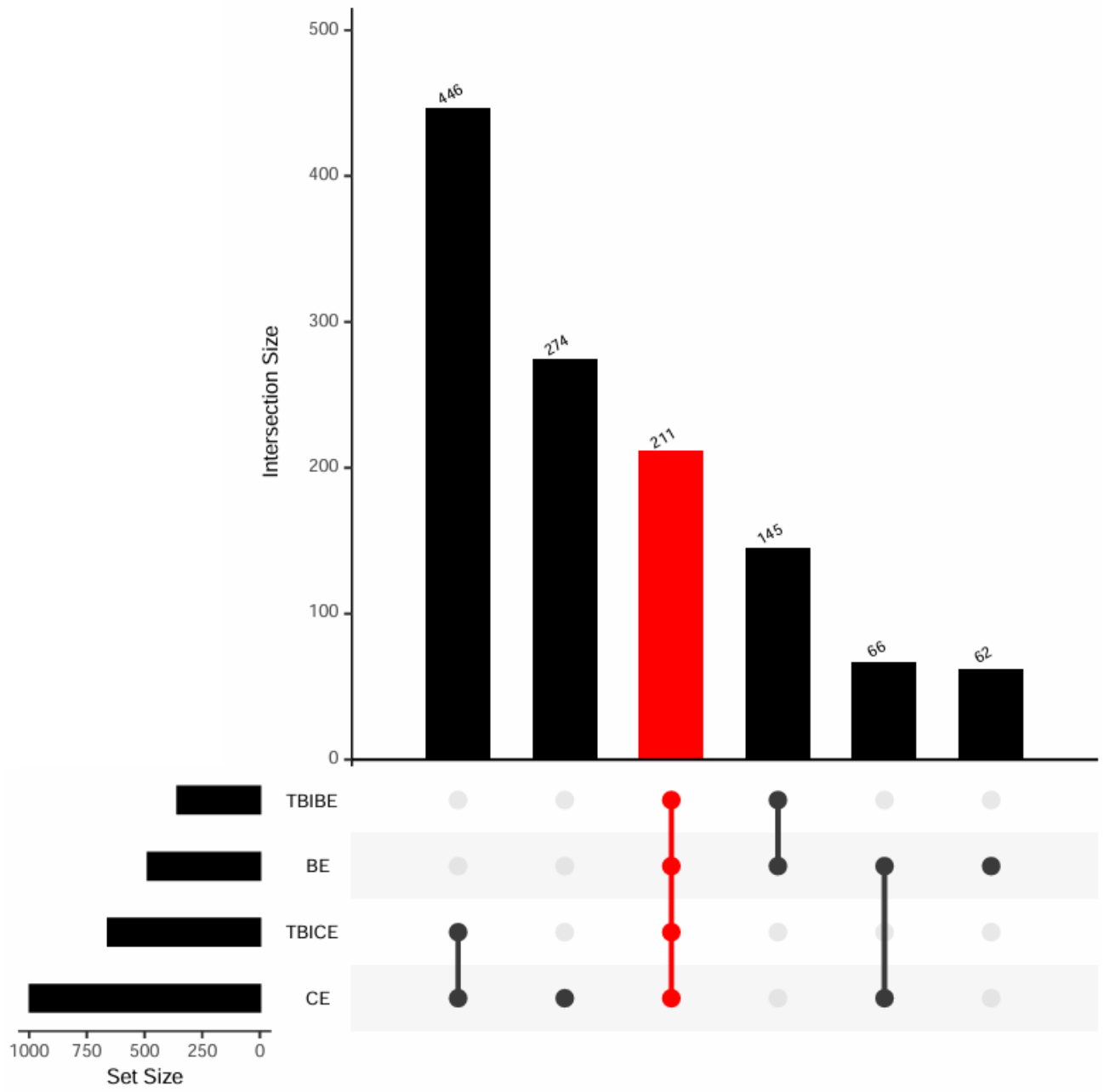

Figure S2. GO enrichment analysis of cerebral edema related genes.

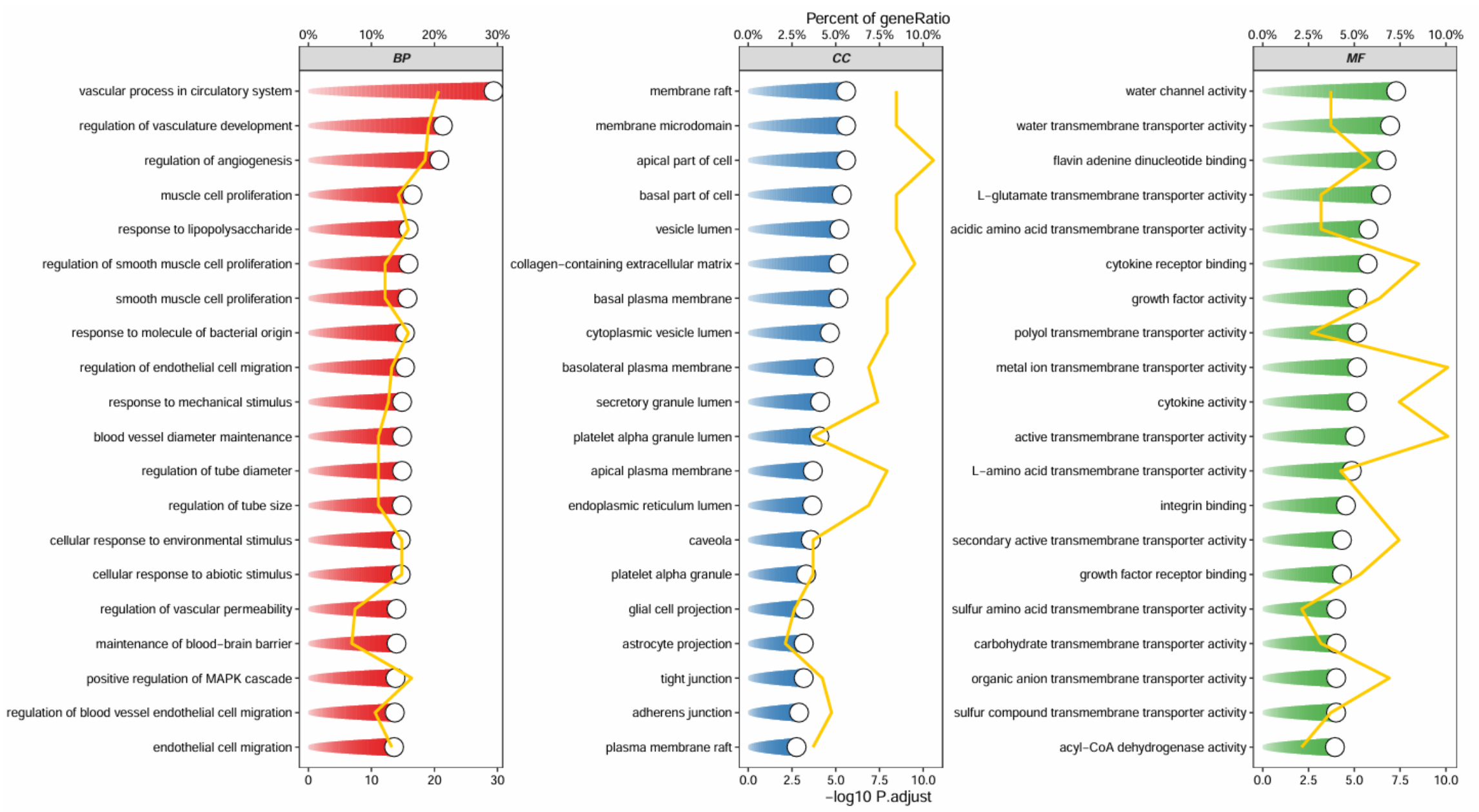

Figure S3. Mfuzz clustering analysis and functional enrichment of genes associated with brain edema at various time points (sham group, TBI-6h, TBI-12h, TBI-1d, TBI-3d, TBI-7d) following TBI.

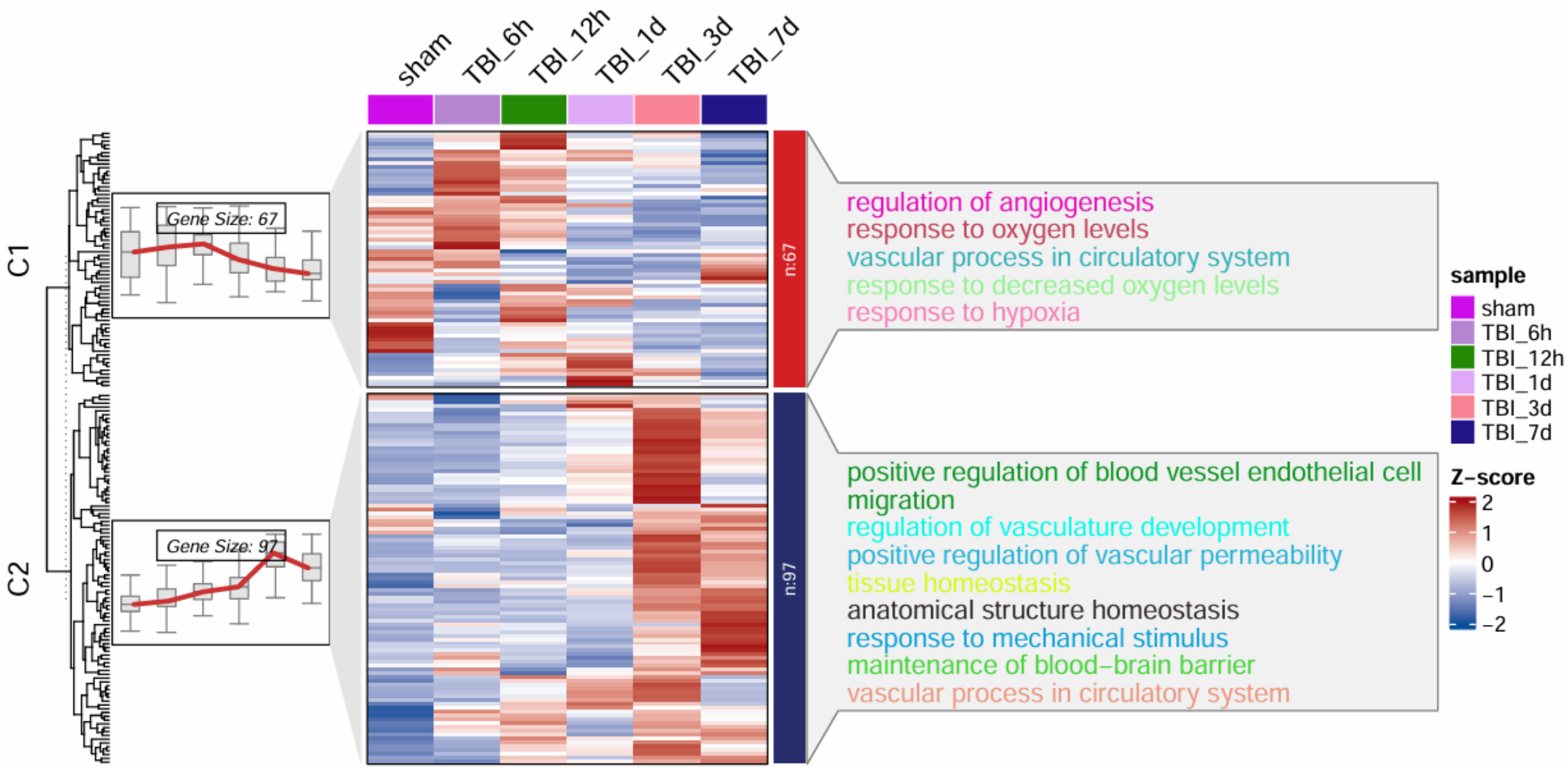

Figure S4. GSVA-based cerebral edema score in sham and TBI animals, and its association with MRI-derived edema volume.

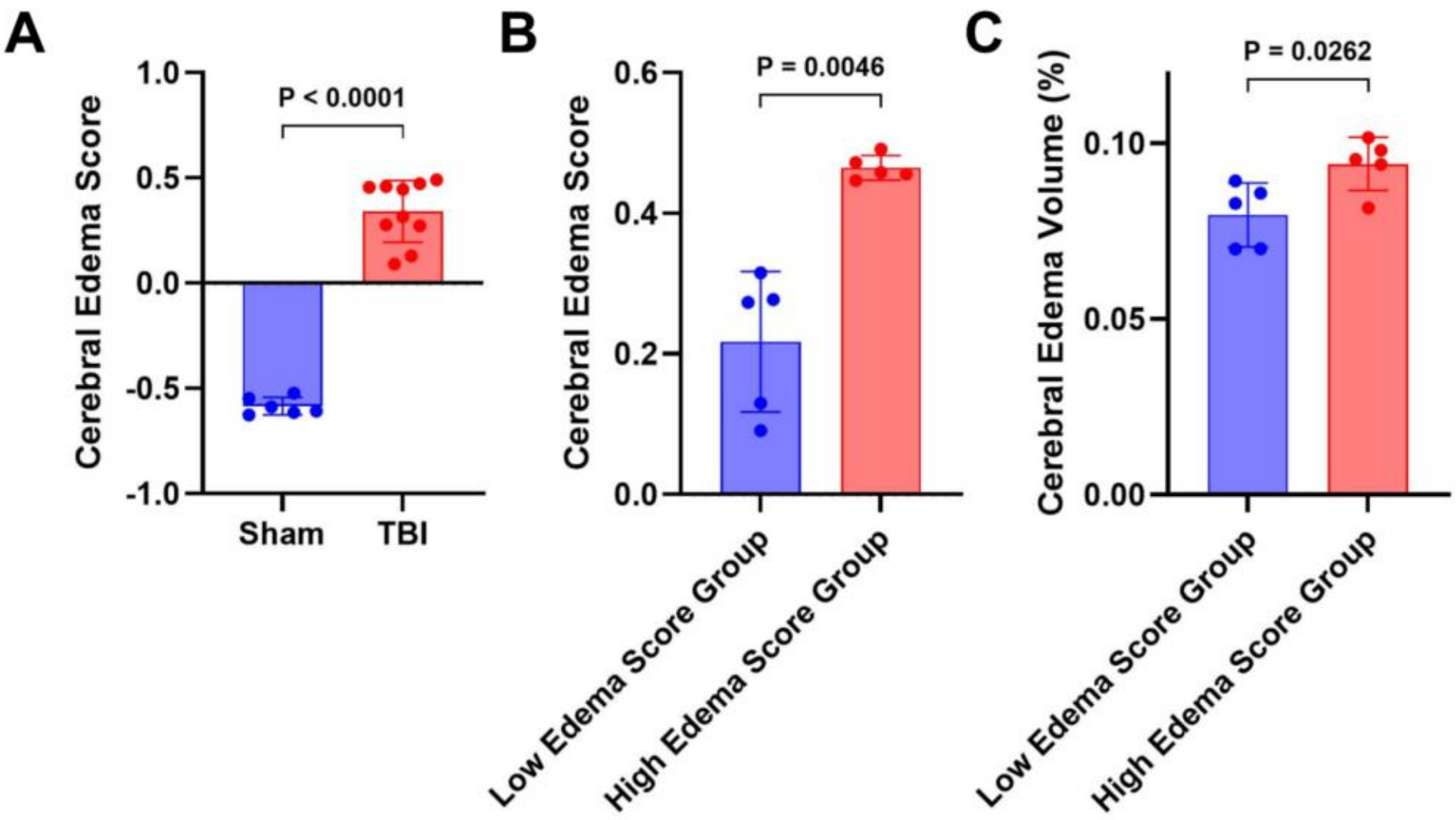

Figure S5. Representative T2-weighted MRI images of Sham, low-score and high-score TBI animals (TBI1 and TBI10).

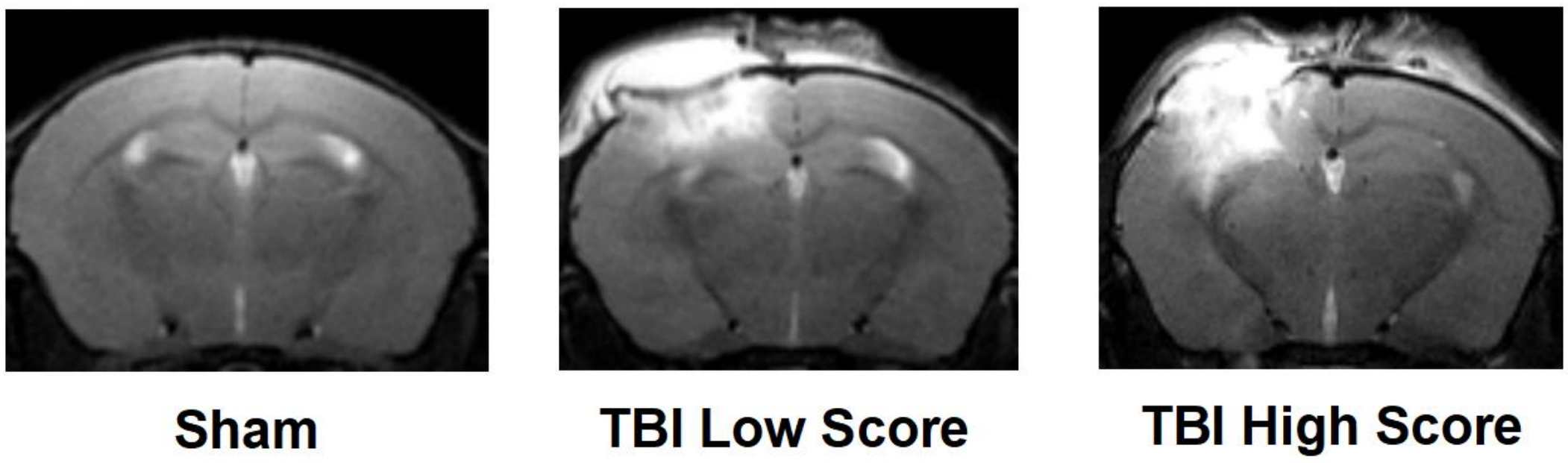

Figure S6. Heatmap of the 97 edema-related gene expression profiles across all individual samples.

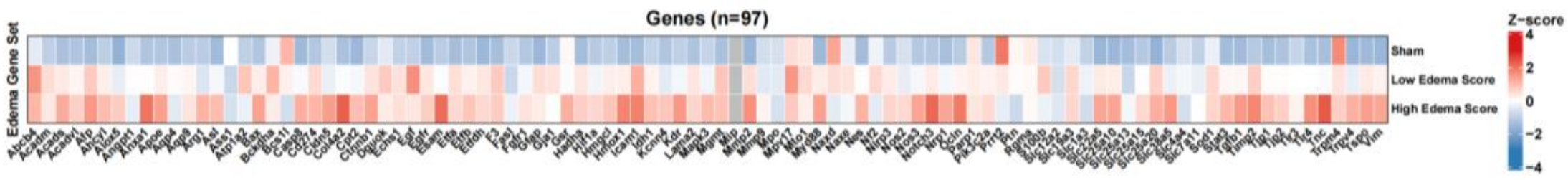

Figure S7. Correlation between GSVA cerebral edema score and MRI-derived edema volume at the individual-animal level (n = 10). TBI1 (low-score) and TBI10 (high-score) are highlighted as representative examples. Linear regression with 95% CI shown.

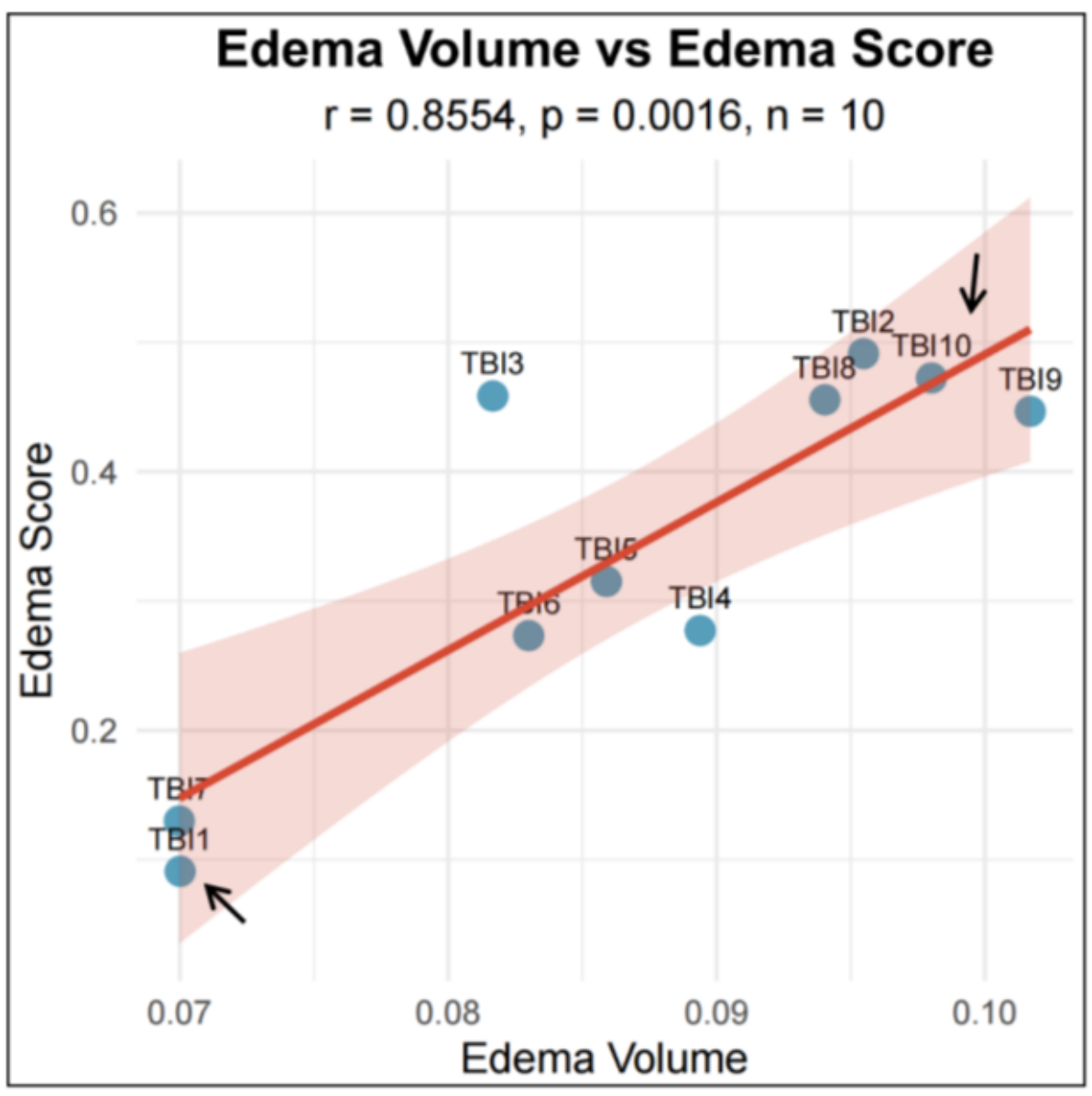

Figure S8. GSVA scores of brain edema gene sets at key time points after TBI.

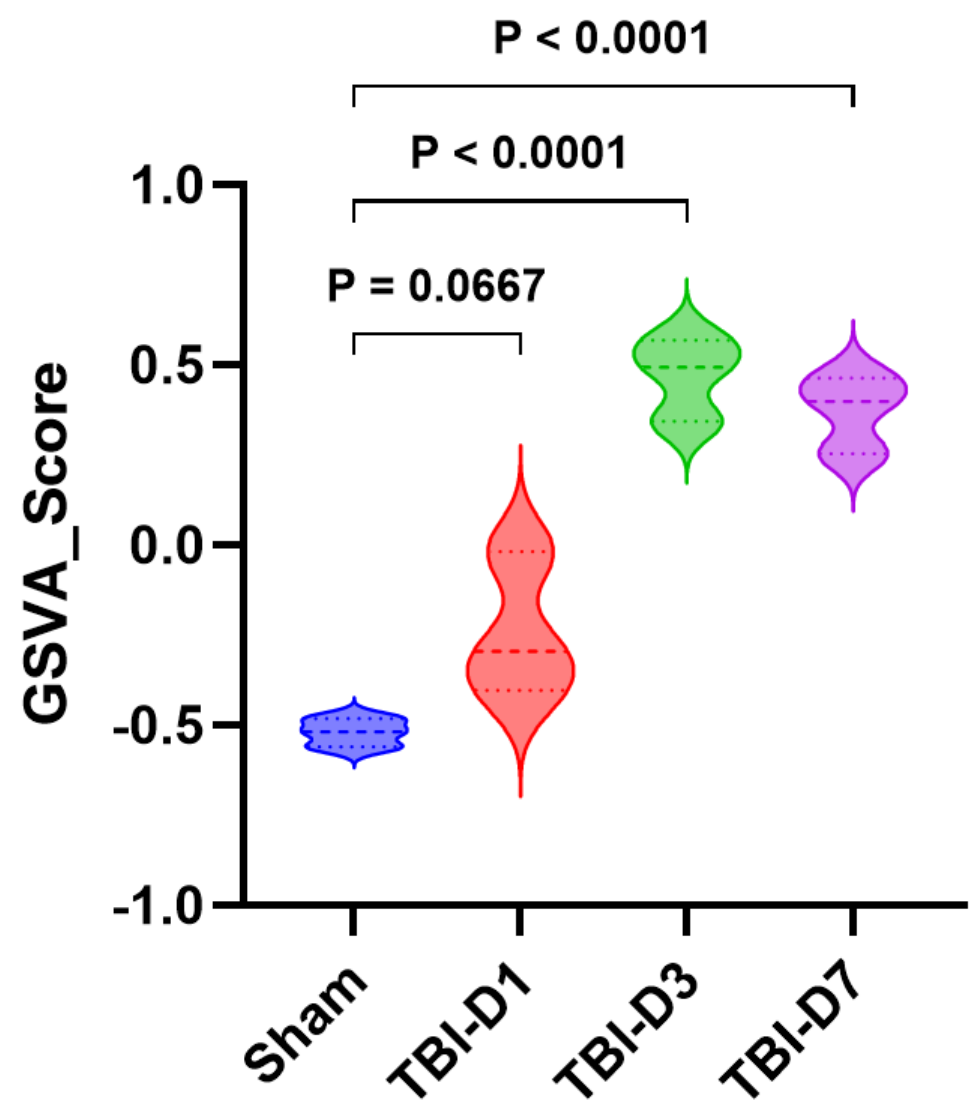

Figure S9. Dot plot of marker genes used for cell population annotation in the GSE175430 dataset.

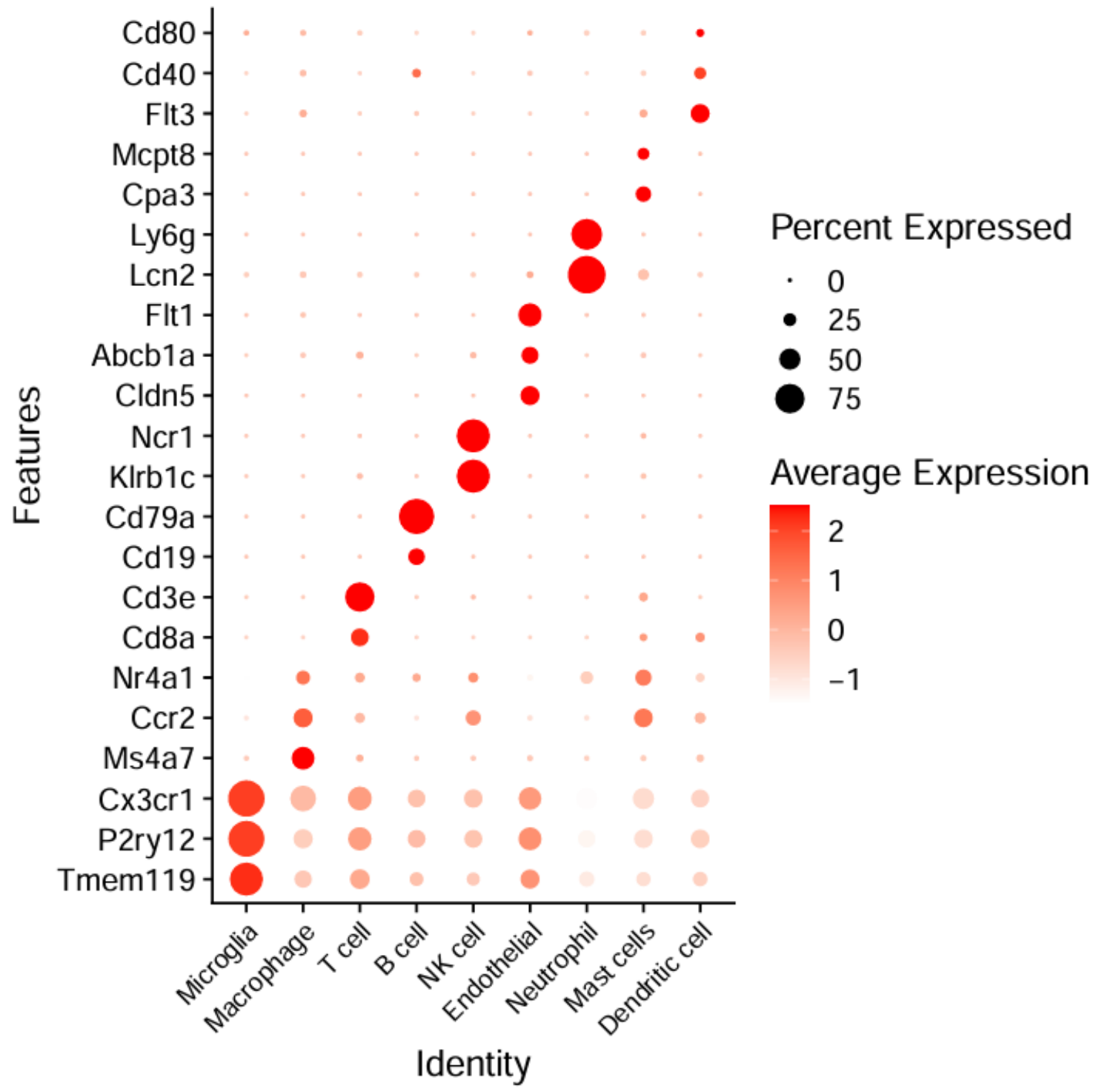

Figure S10. Dot plot of marker genes used for cell population annotation in the GSE266873 dataset.

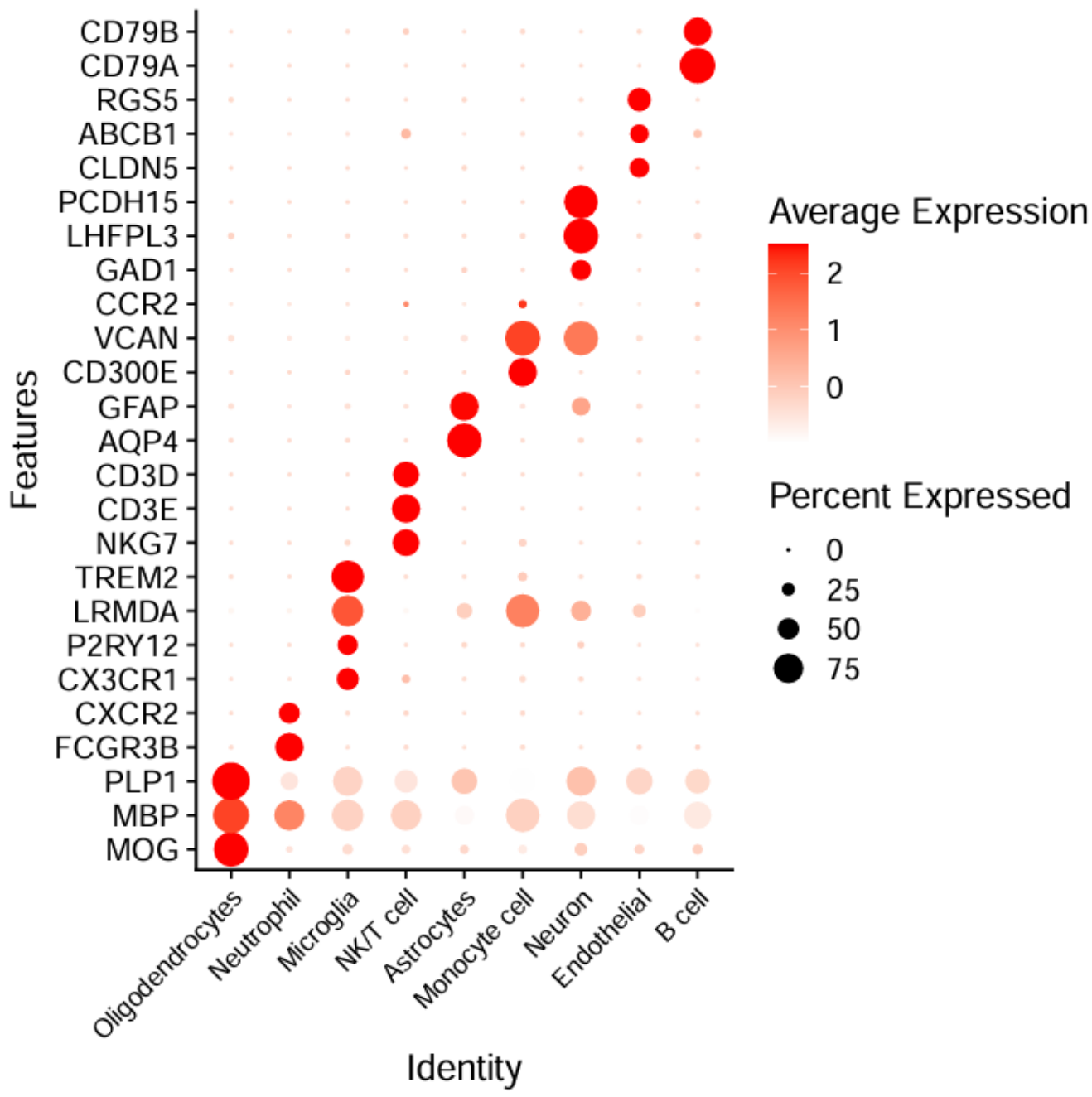

Figure S11. Distribution of the AUCell-based edema-associated score across experimental groups and cell types in GSE175430. The score was increased after TBI. (*, P<0.05; ****, P<0.0001)

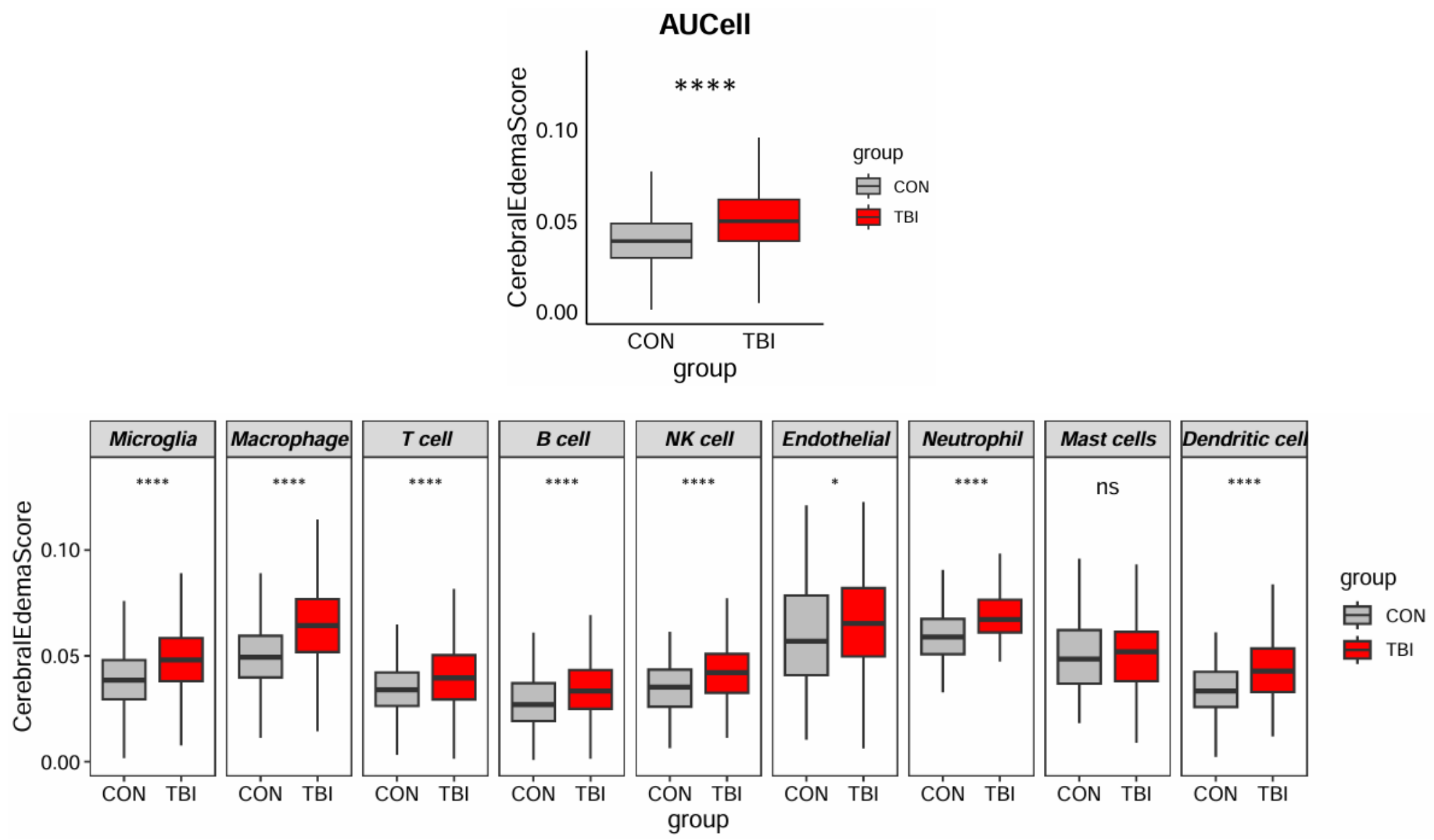

Figure S12. The distribution (left) and proportion (right) of cells with high and low edema scores in the single-cell dataset GSE175430.

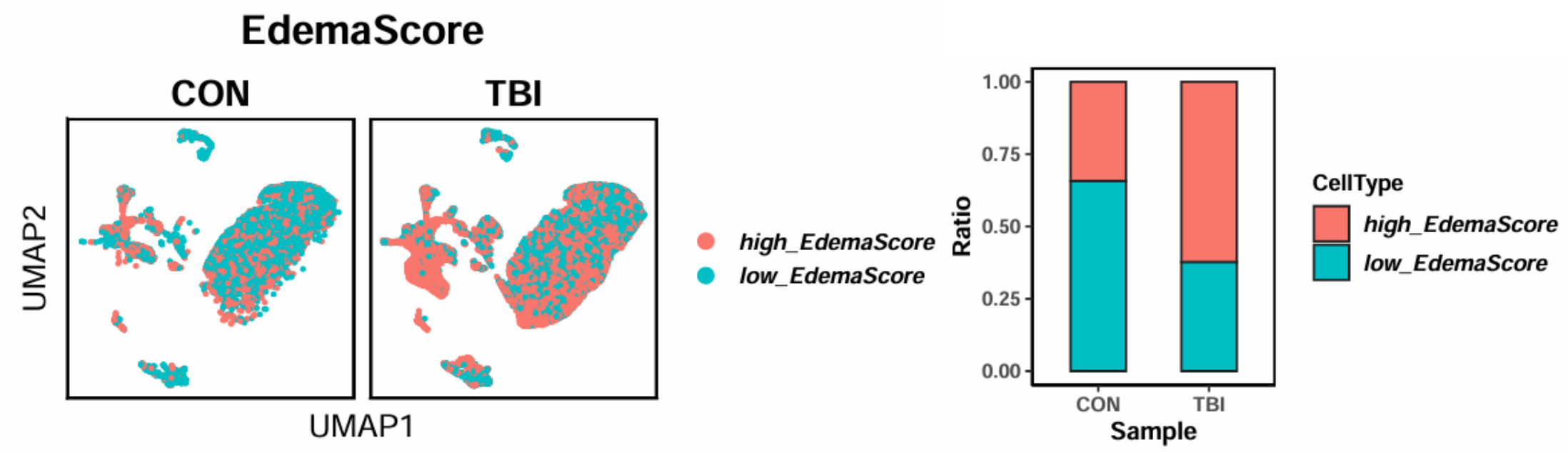

Figure S13. Trajectory analysis (A) and density plot (B) with ACP5-positive macrophages in GSE175430.

A.

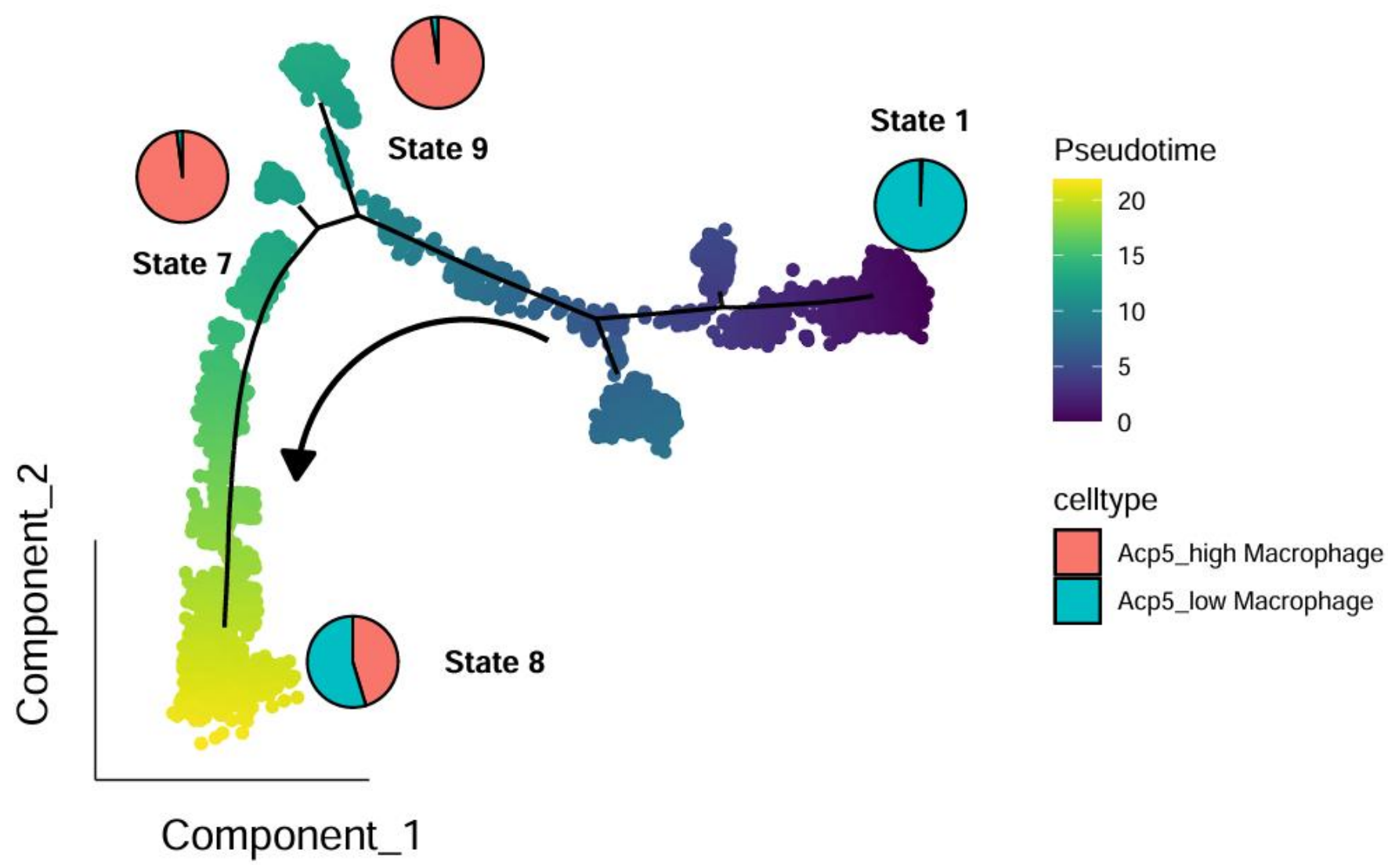

B.

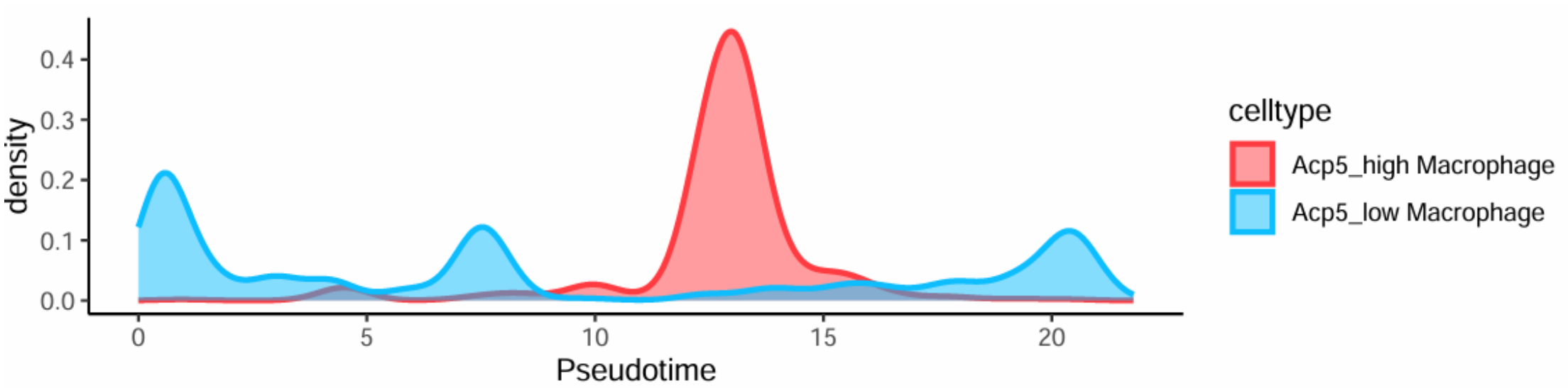

Figure S14. Trajectory analysis (A) and density plot (B) with ACP5-positive monocytes in GSE266873.

A.

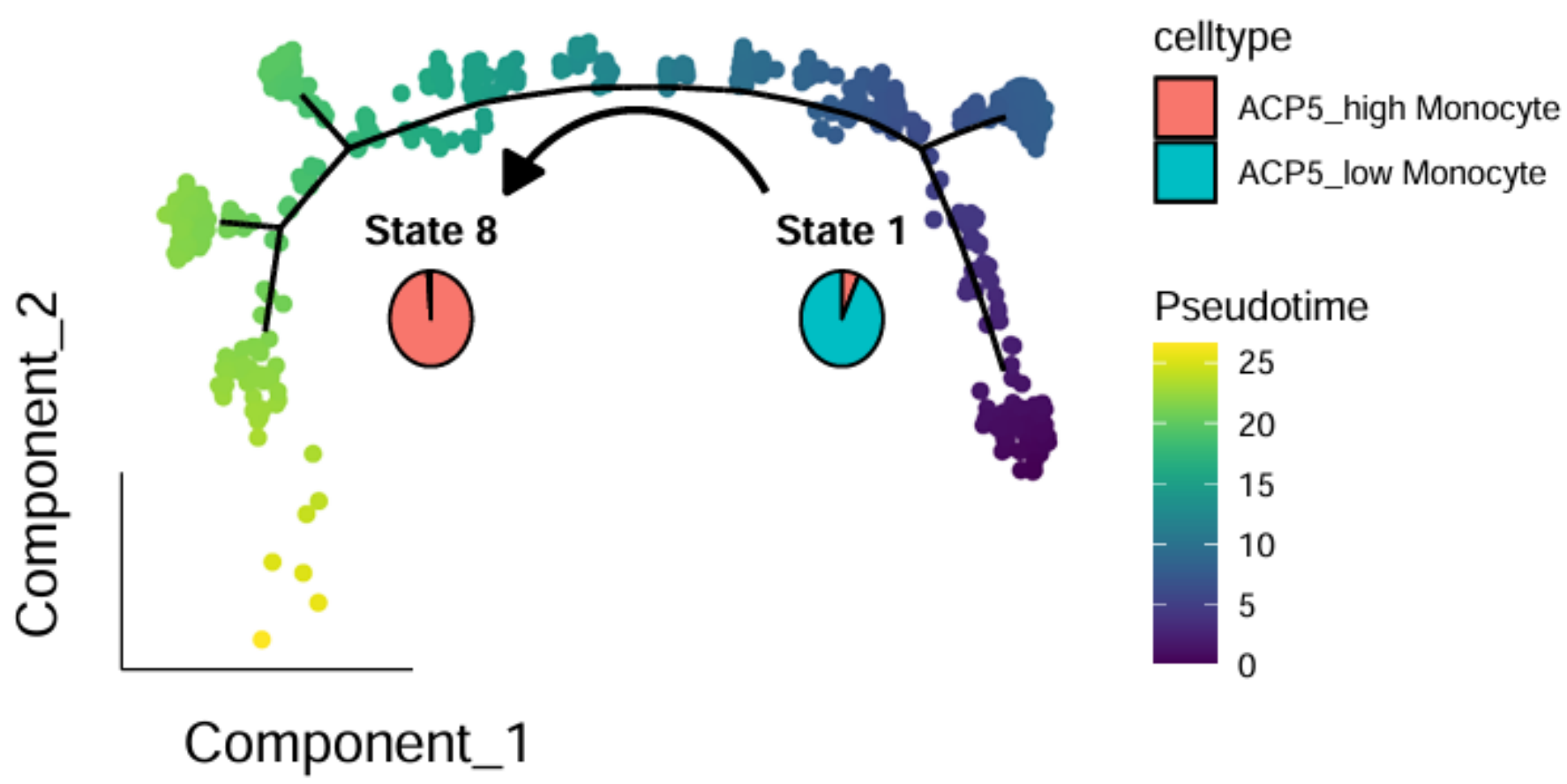

B.

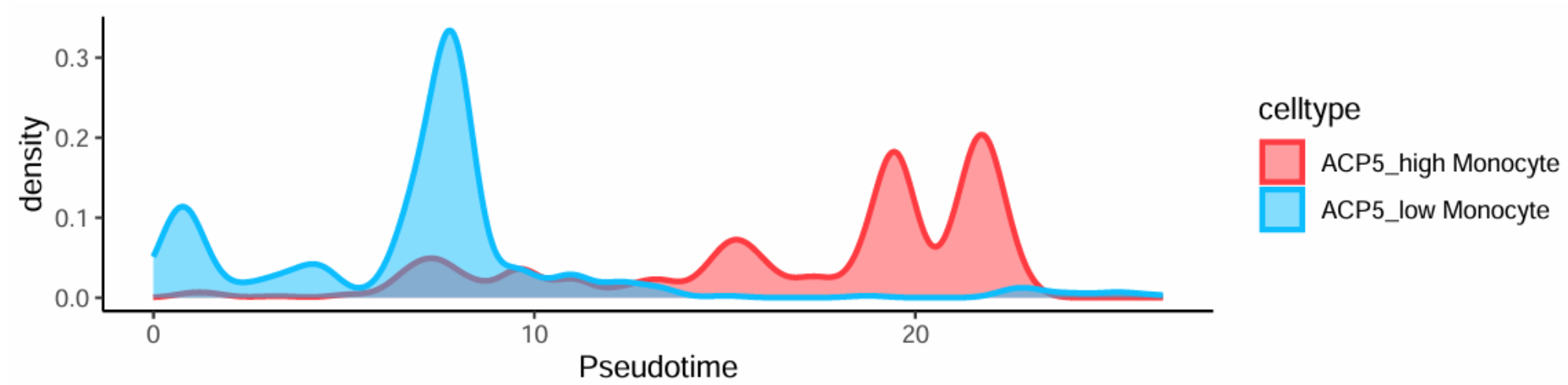

Figure S15. Heatmap of predicted signaling pathways associated with different cell populations in GSE175430.

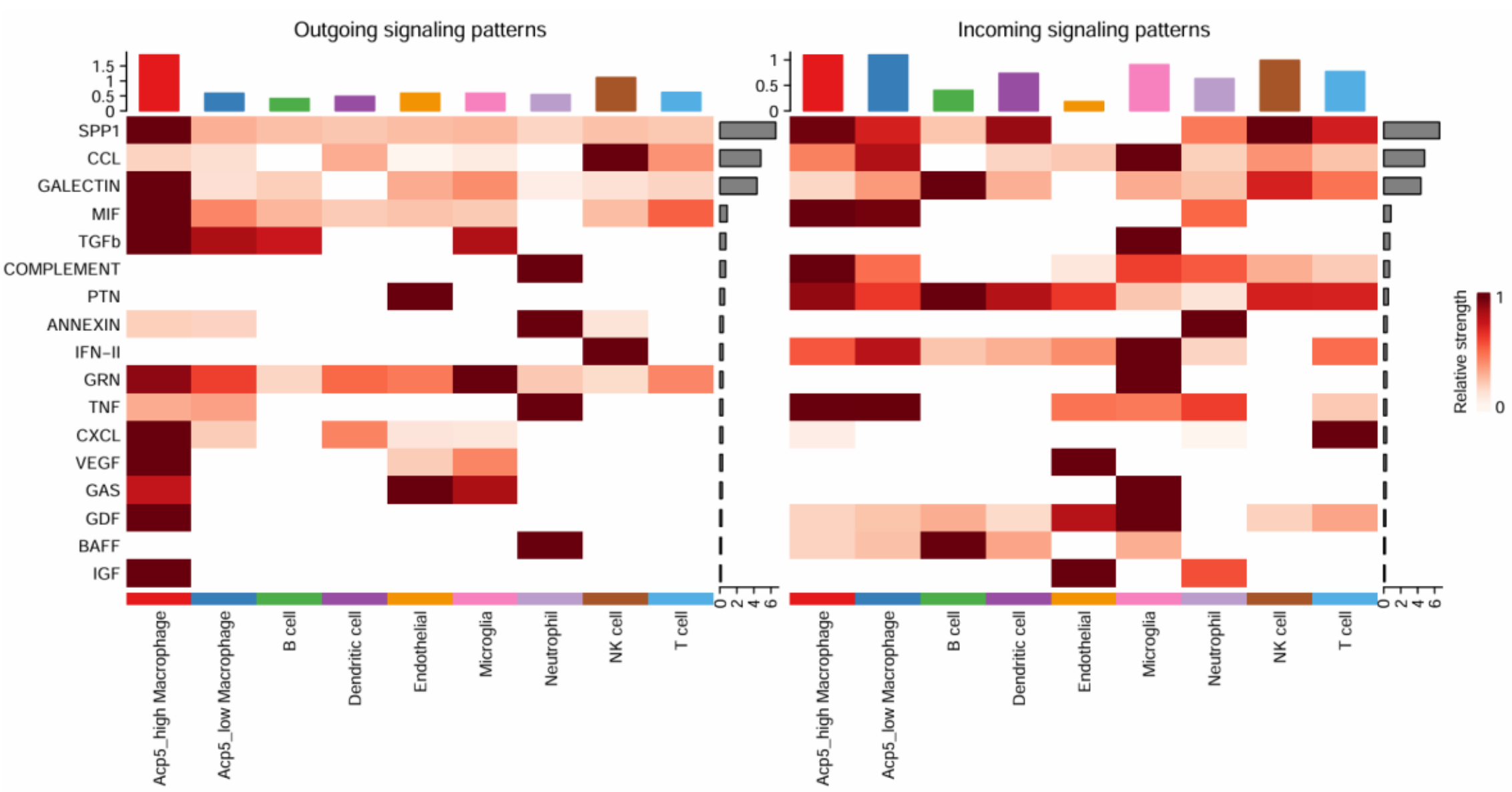

Figure S16. Heatmap of predicted signaling pathways associated with different cell populations in GSE266873.

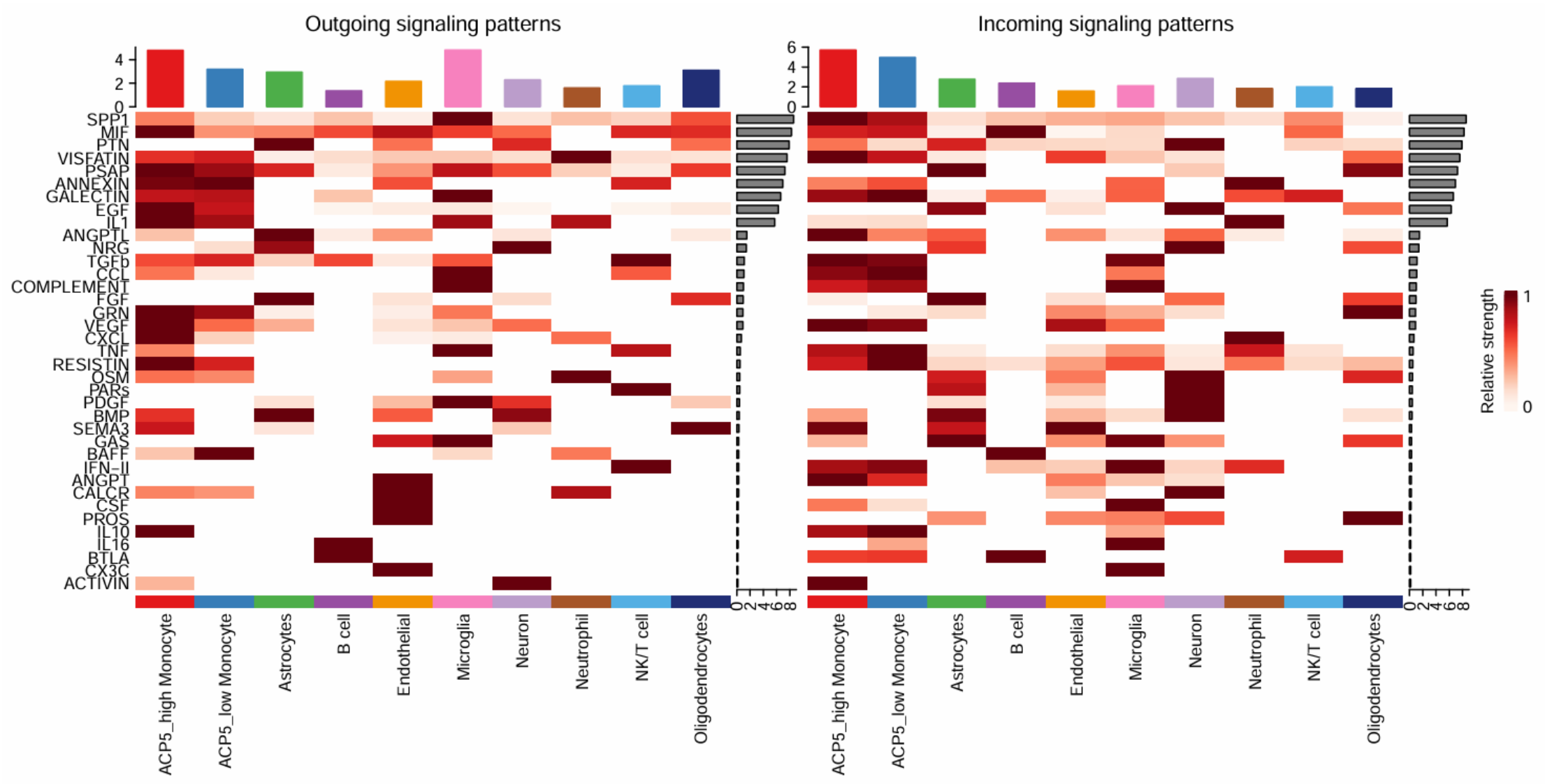

Figure S17. Predicted ligand–receptor interactions inferred by CellChat analysis in GSE175430.

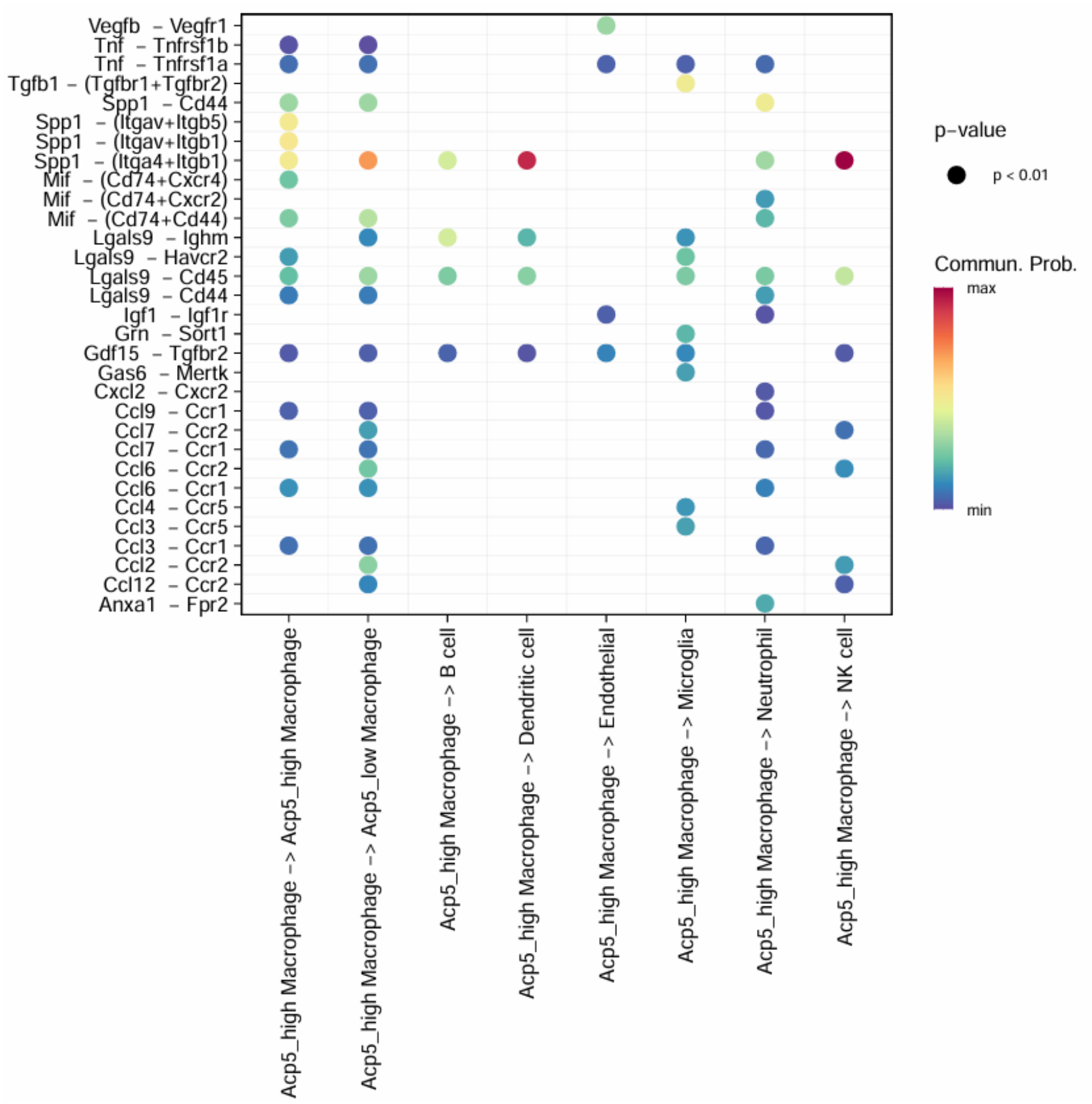

Figure S18. Predicted ligand–receptor interactions inferred by CellChat analysis in GSE266873.

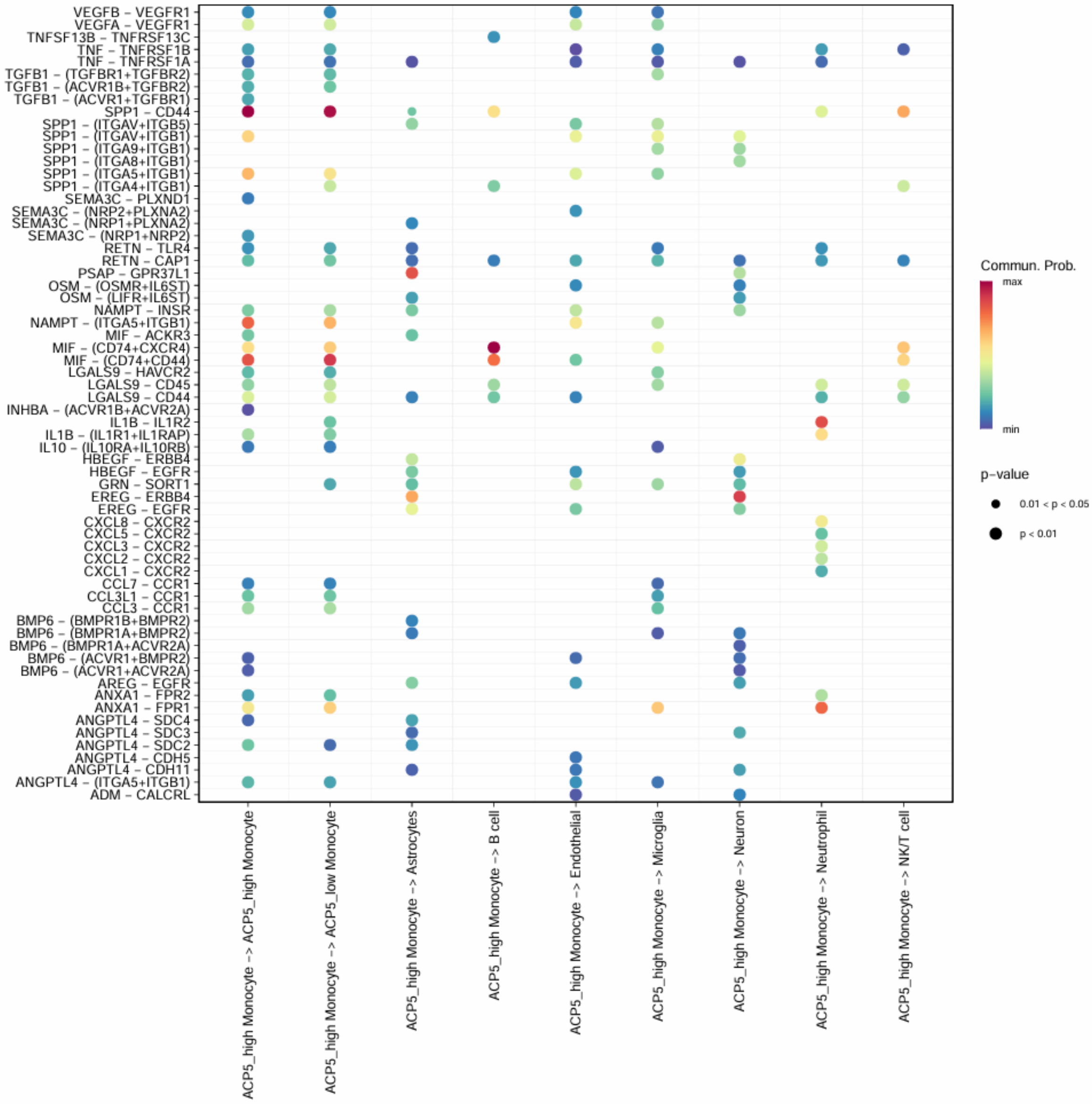

Figure S19. Immunofluorescence staining analysis of peripheral infiltrating macrophages in brain tissue from mice with mild or severe TBI.

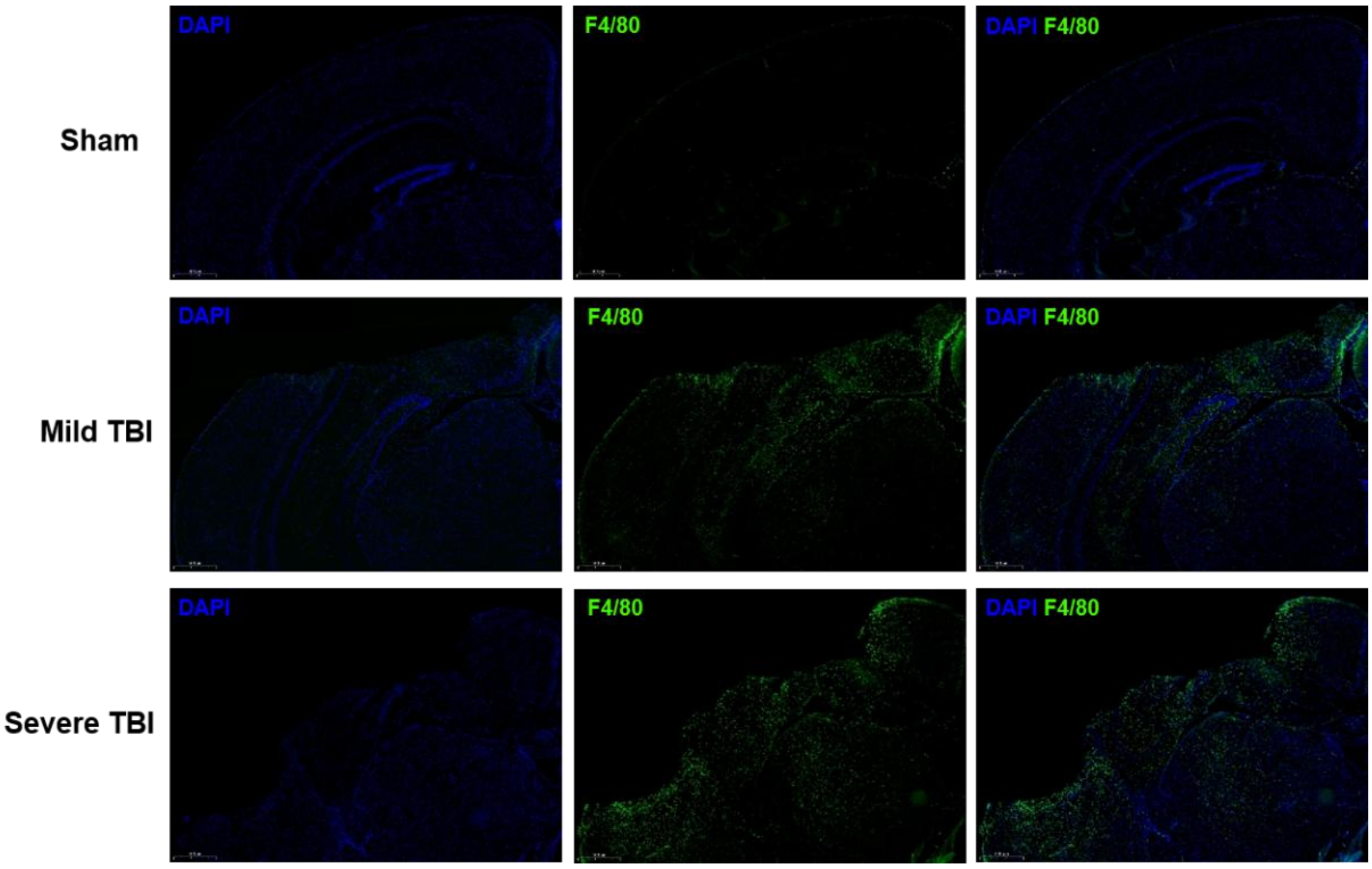

Figure S20. H&E staining of brain tissues of various time points (sham, TBI-12h, TBI-1d, TBI-3d, TBI-7d) following TBI.

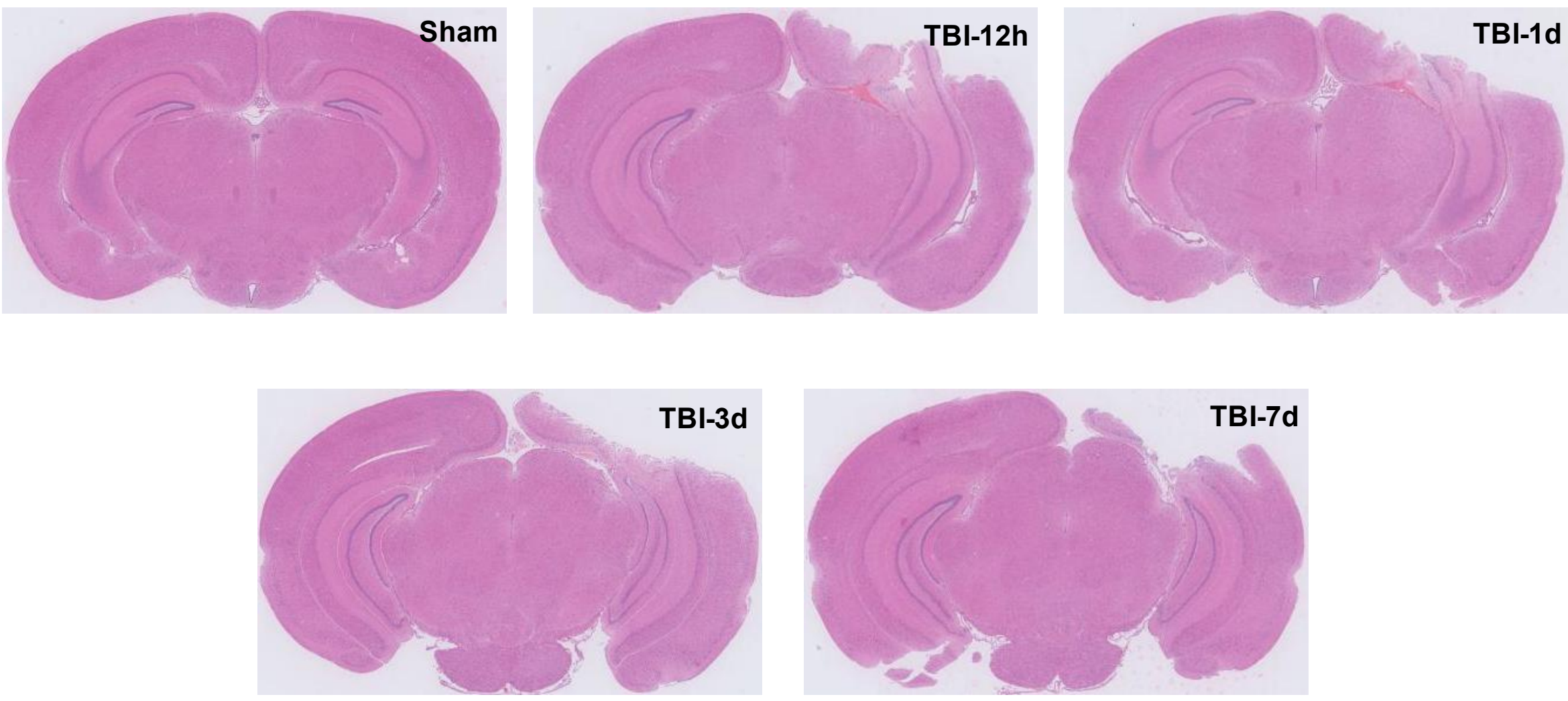

Figure S21. The GSEA results of RNA-seq in the TBI+AubipyOMe group compared to the TBI+Vehicle group.

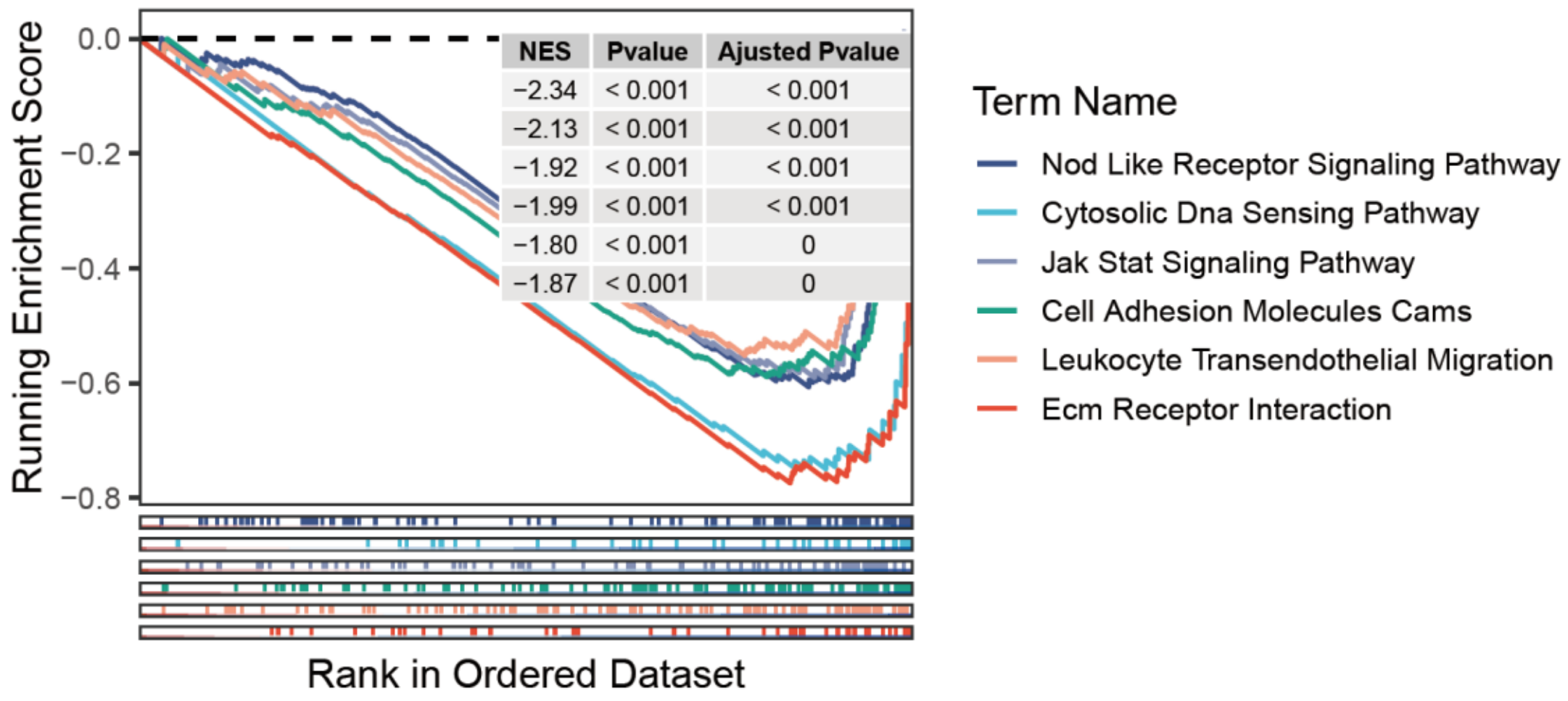

Figure S22. Immune infiltration analysis results of RNA-seq in the TBI+AubipyOMe group and the TBI+Vehicle group (*, P<0.05; **, P<0.01).

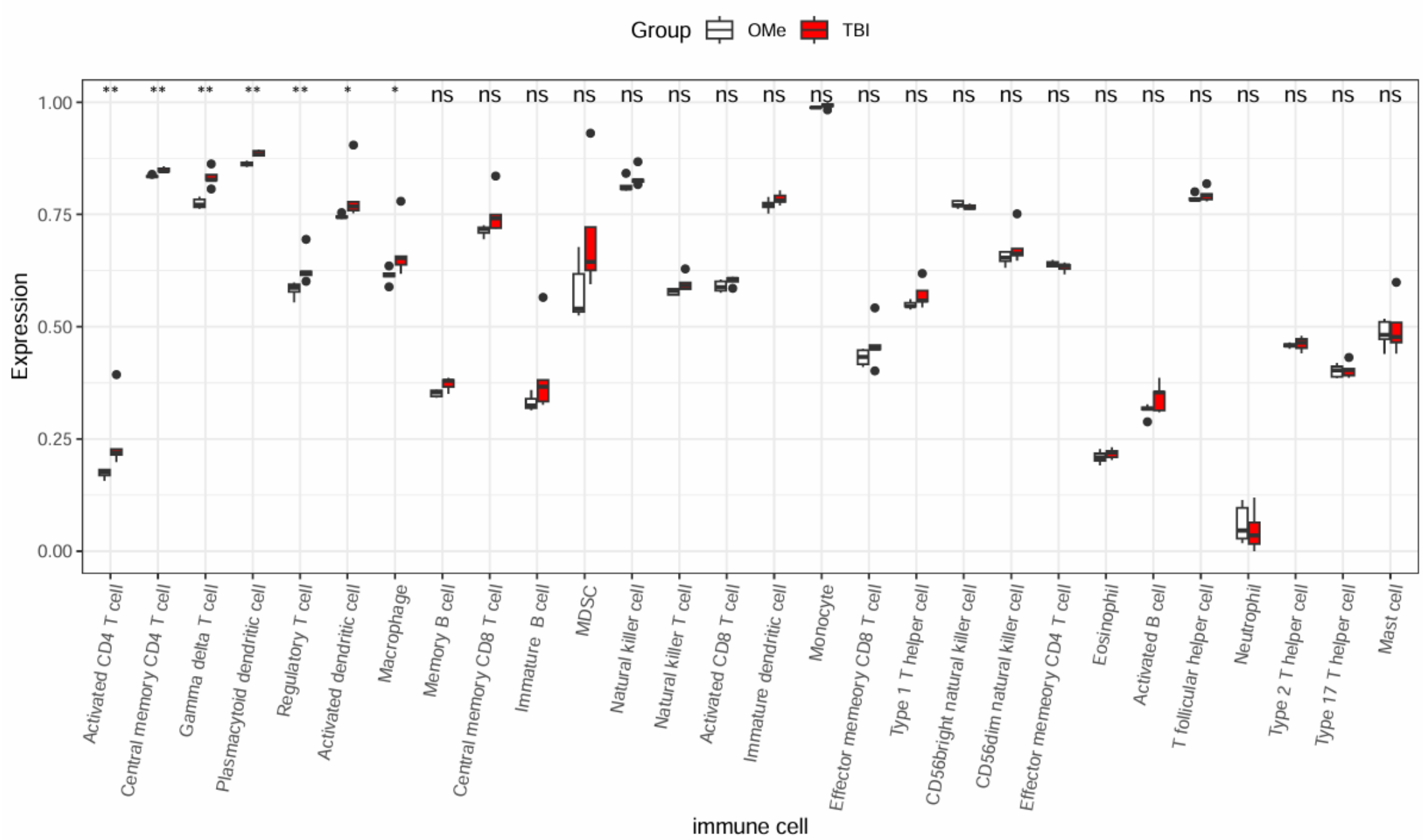

Figure S23. Knockdown efficiency of Acp5 in RAW264.7 cells mediated by siRNA.

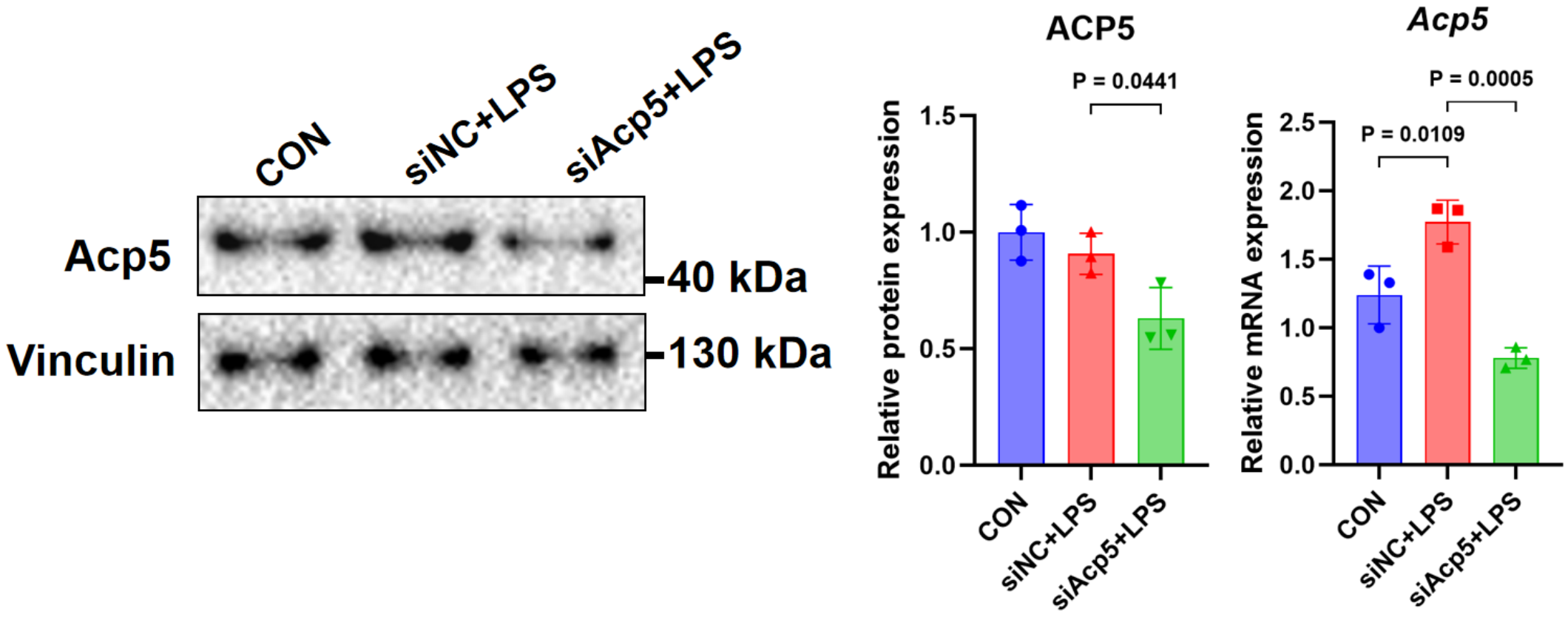

Figure S24. The GSEA results of RNA-seq in the siAcp5+LPS group and the siNC+LPS group.

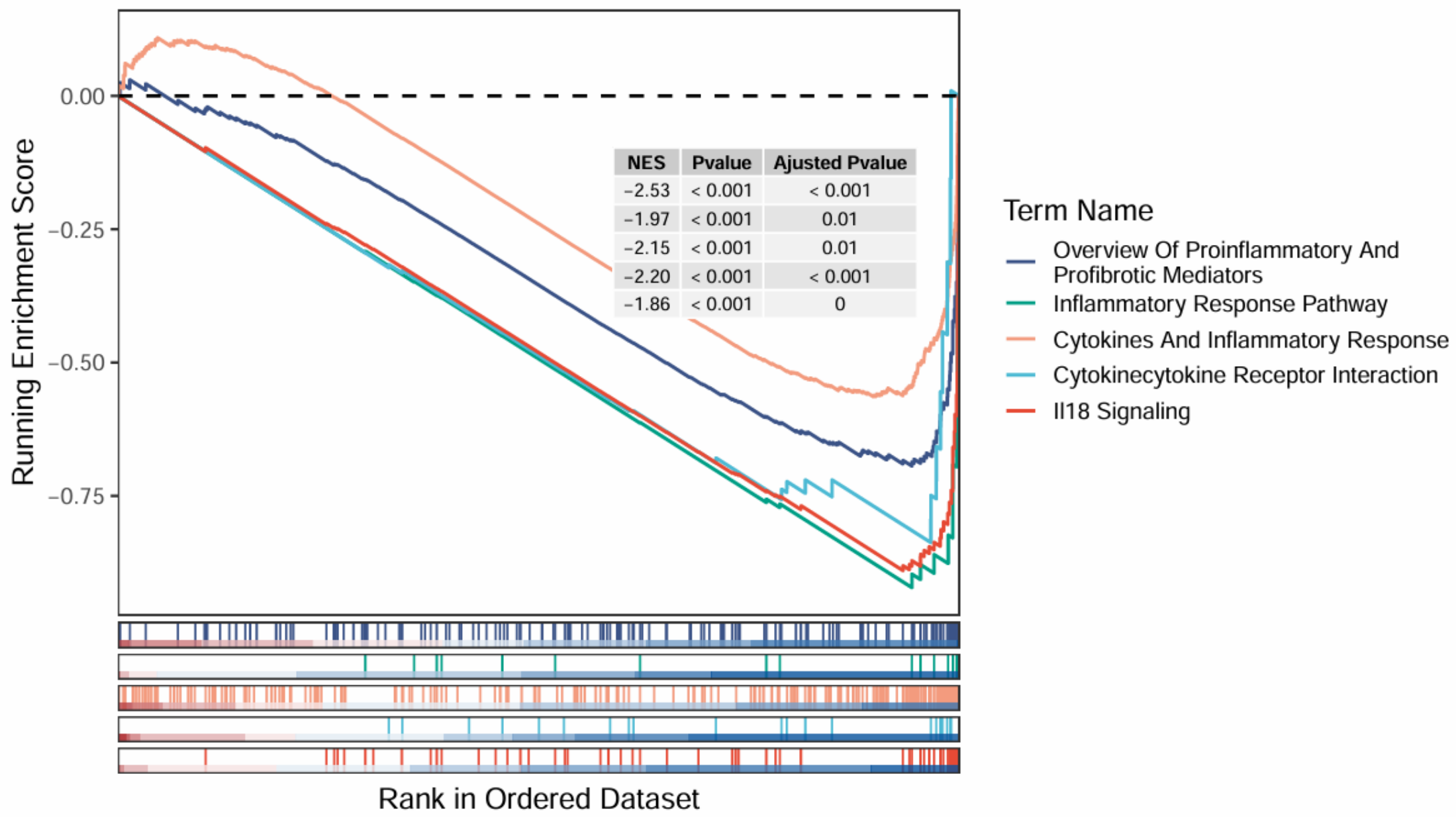

Figure S25. Concentration-dependent effects of AubipyOMe on cell viability, Acp5 expression, and ACP5 enzymatic activity in RAW264.7 cells. A, cell viability; B, *Acp5* mRNA; C, ACP5 protein; D, ACP5 enzymatic activity.

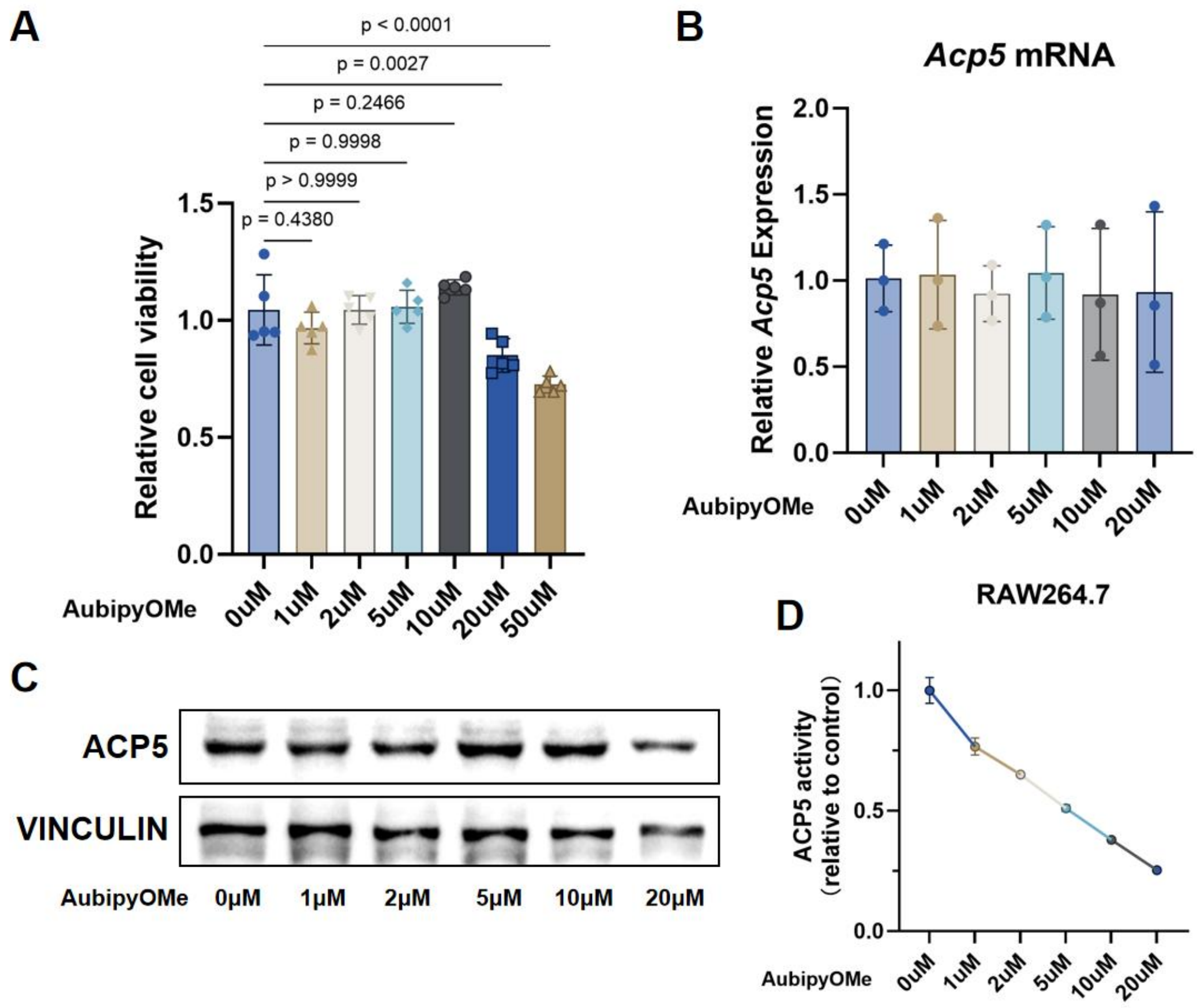

Figure S26. Preliminary hematological (A), biochemical (B), and histopathological safety assessment (C) of AubipyOMe in vivo.

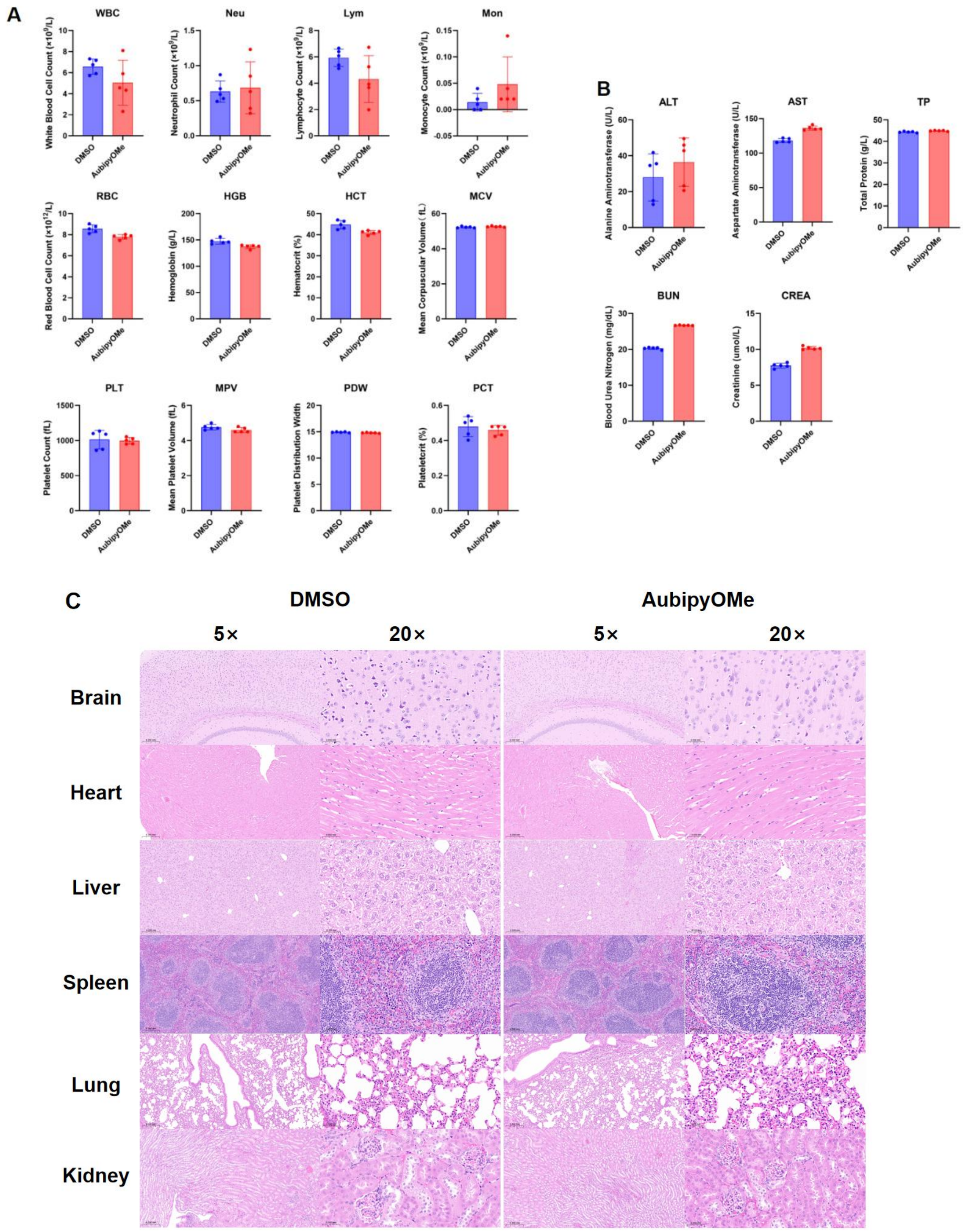

Figure S27. Swimming speed during the Morris water maze probe trial in WT-sham, *Acp5*-KO-sham, WT-TBI, and *Acp5*-KO-TBI mice. Data are presented as the mean ± SD (n = 10).

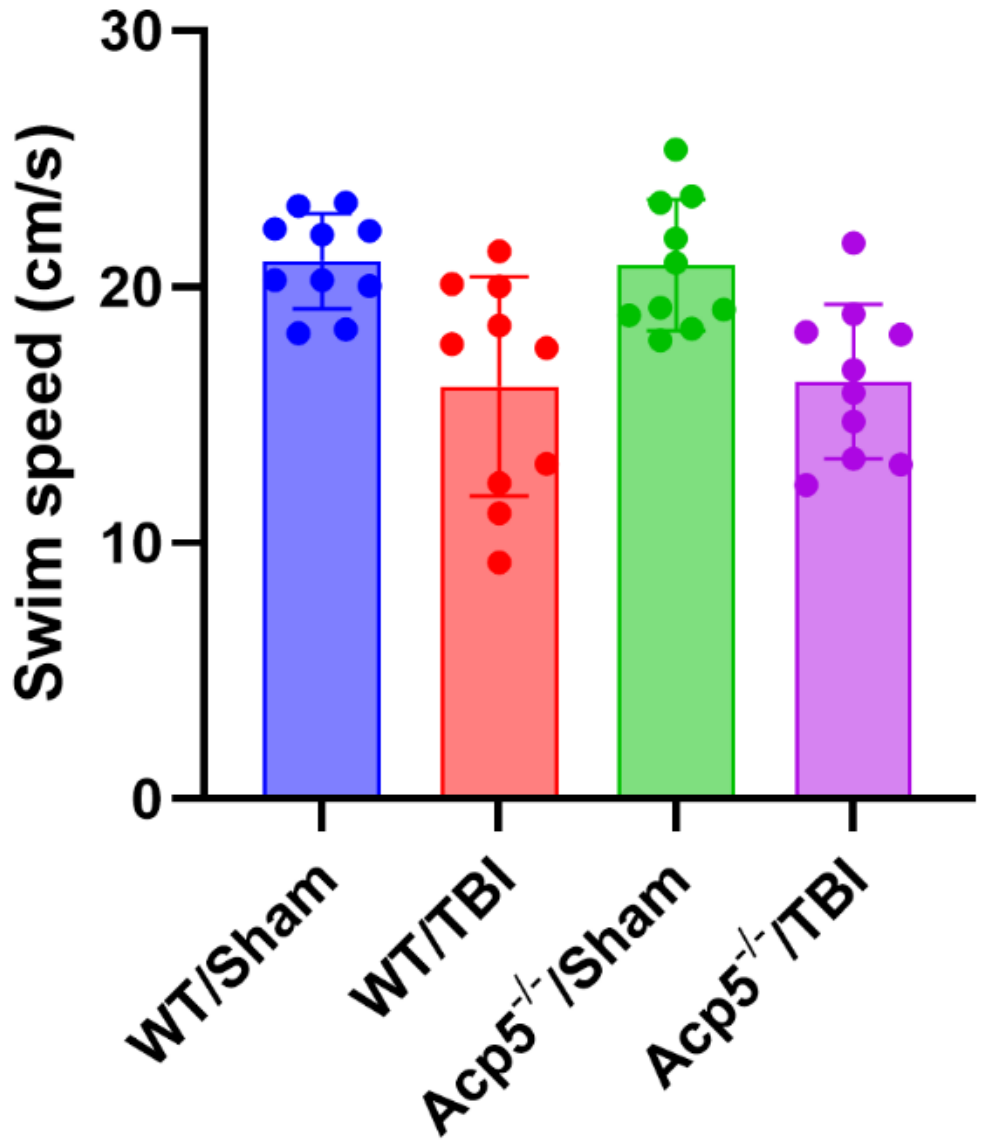

Supplement: Supplementary file 1 [file CTM2-16-e70845-s001.pdf]
